# Single-Cell Dissection of the Immune Microenvironment in Intrahepatic Metastasis of Multifocal Hepatocellular Carcinoma

**DOI:** 10.34133/research.1372

**Published:** 2026-07-27

**Authors:** Yuyan Xu, Cheng Zhang, Zhuocheng Ji, Chang Liu, Lei Cai, Chunming Wang, Hangyu Liao, Yaohong Wen, Luhao Chi, Chang Li, Yucheng Huang, Hongbo Guo, Qing Peng, Mingxin Pan

**Affiliations:** ^1^ Second Department of Hepatobiliary Surgery, General Surgery Center, Guangdong Provincial Research Center for Artificial Organ and Tissue Engineering, Guangzhou Clinical Research and Transformation Center for Artificial Liver, Institute of Regenerative Medicine, Zhujiang Hospital, Southern Medical University, Guangzhou, China.; ^2^ School of Biomedical Sciences and Engineering, South China University of Technology, Guangzhou, China.; ^3^ PCFM Lab of Ministry of Education, School of Materials Science and Engineering, Sun Yat-sen University, Guangzhou, China.; ^4^ Neurosurgery Center, National Key Clinical Specialty, Engineering Research Center of Diagnostic and Therapeutic Technology and Devices for Cerebrovascular Diseases in Ministry of Education, Guangdong Provincial Key Laboratory on Brain Function Repair and Regeneration, Zhujiang Hospital Institute for Brain Science and Intelligence, Zhujiang Hospital, Southern Medical University, Guangzhou, China.; ^5^ Central Laboratory of The Second Affiliated Hospital, School of Medicine, The Chinese University of Hong Kong, Shenzhen & Longgang District People’s Hospital of Shenzhen, Shenzhen, China.

## Abstract

Intrahepatic metastasis in multifocal hepatocellular carcinoma is associated with poor prognosis and therapeutic resistance, yet the immune mechanisms driving disease progression remain unclear. Here, we analyzed genetic and immune differences between primary tumors and intrahepatic metastatic lesions using sequencing approaches and spatial validation methods. We found that metastatic lesions shared key genomic features with primary tumors but exhibited a distinct immunosuppressive environment enriched in myeloid and T cell populations. In particular, a subset of macrophages expressing glycoprotein nonmetastatic melanoma protein B (GPNMB) was consistently enriched in metastatic niches across multiple independent cohorts. These macrophages were spatially colocalized with CD8^+^ T cells exhibiting features of terminal exhaustion. Mechanistically, integrated multiomics and functional analyses revealed that GPNMB overexpression triggers lipid metabolic rewiring via the phosphatidylinositol 3-kinase/AKT-cyclooxygenase-2 cascade, leading to elevated prostaglandin E2 secretion, which directly suppresses CD8^+^ T cell cytotoxicity. Specific silencing of this subset using a dual-targeted, lipid-polymer nanoparticle (APL_siGpnmb_) effectively reversed T cell exhaustion, inhibited metastasis, and synergized with anti-programmed death 1 immunotherapy in mouse models without inducing systemic toxicity. These findings identify GPNMB-positive macrophages as key metabolic and immune regulatory hubs, suggesting that targeting the GPNMB–prostaglandin E2 axis provides a promising precision therapeutic strategy for intrahepatic metastasis in multifocal hepatocellular carcinoma.

## Introduction

Hepatocellular carcinoma (HCC) imposes a considerable global health burden as a primary driver of cancer-linked mortality [1]. At initial diagnosis, approximately 50% to 75% of HCC cases present with multiple lesions. Intriguingly, many patients diagnosed with a solitary tumor via preoperative imaging are found to harbor occult multifocal lesions upon posttransplantation pathological examination [2]. These findings imply that the actual incidence of multifocal HCC (mHCC) may be much higher than clinically estimated. The treatment outcomes for mHCC are often poor, with a postsurgical recurrence rate exceeding 70%. mHCC is broadly categorized into 2 distinct clonal types: multicentric occurrence (MO) and intrahepatic metastasis (IM), with IM accounting for approximately 40% to 70% of mHCC cases [3–5]. MO-mHCC, although of independent clonal origin, is associated with a relatively lower rate of tumor invasion and recurrence [6,7]. In contrast, IM-mHCC is characterized by aggressive dissemination, frequent postoperative recurrence, and resistance to systemic therapies [8]. Consequently, developing precise and effective therapeutic interventions for IM-mHCC has become a major clinical priority.

Currently, the management of IM-mHCC faces a double dilemma: the technical difficulty of achieving curative resection and pervasive therapeutic resistance that compromises conventional treatments [9–11]. Emerging evidence suggests that the tumor immune microenvironment (TIME) orchestrates this resistance by promoting the functional exhaustion in effector immune cells. Single-cell RNA sequencing (scRNA-seq) technologies have recently enabled a deeper understanding of the complexity of the TIME [12–14]. Yang et al. [15] revealed substantial genomic and microenvironmental heterogeneity in mHCC of different origins, with IM-mHCC exhibiting stronger immunosuppressive characteristics. Zhou et al. [16] identified C5aR-positive macrophages as key drivers of immunosuppressive remodeling in HCC portal vein tumor thrombi, suggesting that specific myeloid subsets may orchestrate immune evasion in metastatic niches. However, the precise identity of the key immunosuppressive cellular subsets in the IM, their functional mechanisms, and their specific crosstalk with effector immune cells remain elusive. Therefore, clarifying the roles of immune cell subsets in the TIME of IM-mHCC is essential for the development of effective treatments.

This study was designed to investigate the complex TIME heterogeneity within different IM-mHCC lesions via single-cell transcriptomic profiling. We sought to clarify the spatial distribution and functional characteristics of immune cell subsets. By elucidating the specific functions of these immune cell subsets in the TIME of IM-mHCC, this work offers a foundation upon which effective treatments can be developed. We demonstrate the role of glycoprotein nonmetastatic melanoma protein B (GPNMB)-positive macrophages (GPNMB^+^ Mph) in promoting tumor progression, suggesting their utility as novel therapeutic targets in IM-mHCC.

## Results

### Genetic heterogeneity in PL and IM

**Fig. 1.**
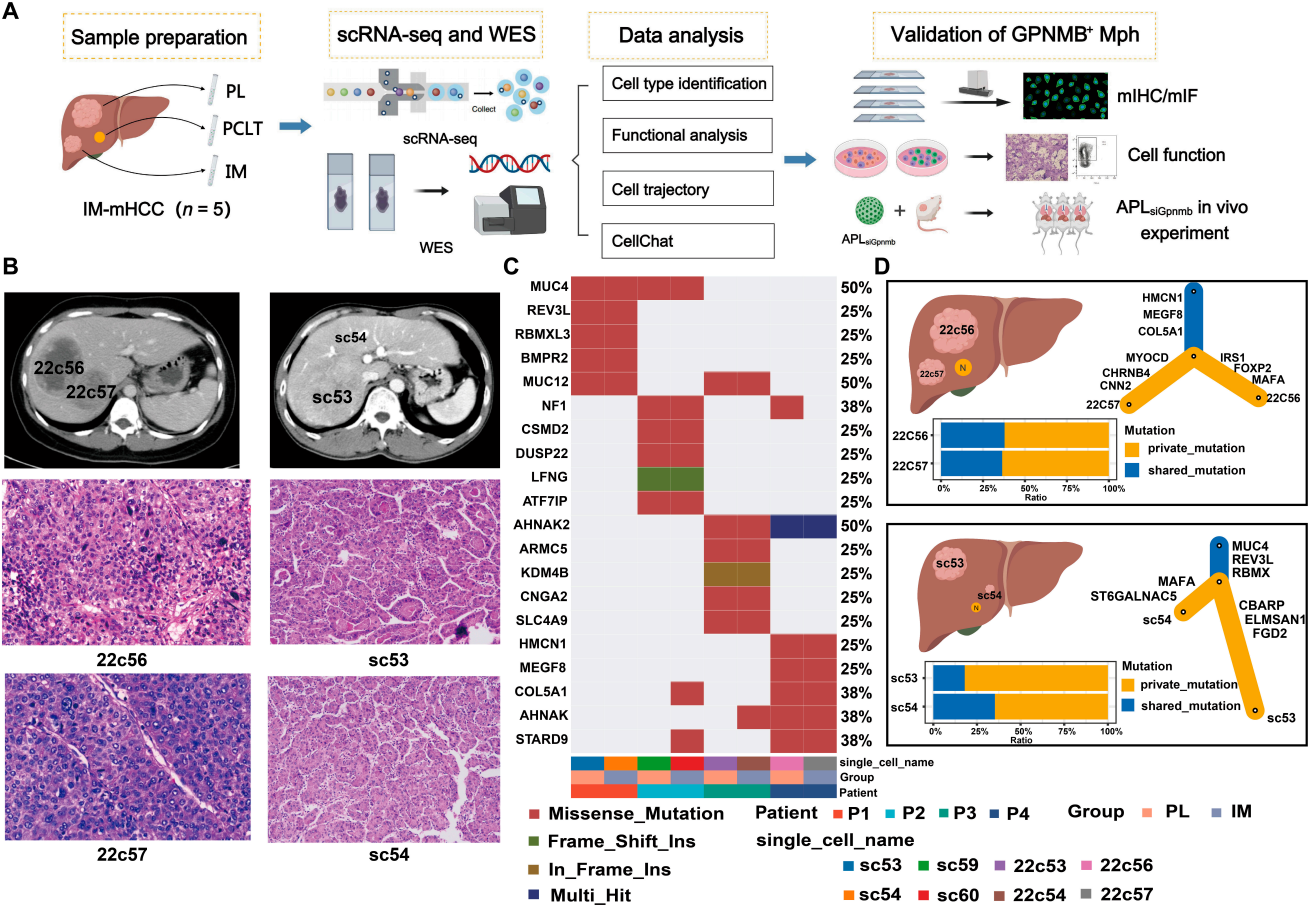
Genomic landscape of intrahepatic metastatic multifocal hepatocellular carcinoma (IM-mHCC). (A) Simplified flowchart of the procedures for tissue sample collection, single-cell RNA sequencing (scRNA-seq) and whole-exome sequencing (WES) processing, data analysis, and experimental validation. (B) Representative case of IM-mHCC showing computed tomography (CT) imaging and histopathological features. (C) Oncoprint of significantly mutated genes identified by WES in the 4 IM-mHCC patients. (D) Phylogenetic reconstructions and spatial maps illustrating the proportions of ubiquitous (shared) versus region-specific (private) somatic variants across multiple tumor sites for each individual with IM-mHCC.

To explore the heterogeneity of the TIME between primary lesion (PL) and IM of IM-mHCC, paired tissue samples from 5 treatment-naïve patients with IM-mHCC were collected for scRNA-seq (Fig. 1A). Initially, patients were clinically diagnosed with IM-mHCC based on integrated imaging and pathological criteria [17]: (a) Each patient had at least 2 independent tumors that were mostly distributed in the same liver lobe or adjacent segments. (b) Contrast-enhanced computed tomography showed similar enhancement patterns. (c) Postoperative pathological reports indicated similar pathological types of lesions (such as pathological differentiation grade and subtypes), most of which were positive for microvascular invasion or satellite nodules. (d) HCC markers (alpha fetoprotein and protein induced by vitamin K absence-II) were elevated, reflecting a high tumor burden and stronger invasiveness. (e) Early recurrence within 2 years postsurgery was observed in all 5 patients, consistent with micrometastasis and corroborating the diagnosis of IM-mHCC (Fig. 1B, Fig S1A, and Table 1).

**Table 1.** Baseline clinicopathological profiles of the IM-mHCC cohort

Patient ID	Lesion no.	Age (≥40)	HBV	Tumor size (≥5 cm)	BCLC	MVI	Satellite nodules	AFP	PIVKA-II	CA199	CEA	RFS
ZJ001	22c56(PL) 22c57(IM) 22c58(PCLT)	Yes	Yes	Yes	B	Yes	No	37.8	1,429.45	2	5.1	10
ZJ002	sc53(PL) sc54(IM) sc55(PCLT)	Yes	Yes	Yes	B	No	Yes	607	1,604.28	49.1	4.3	15
ZJ003	sc59(PL) sc60(IM) sc61(PCLT)	Yes	Yes	No	B	Yes	Yes	1,205	17,395.25	19.3	3.1	13
ZJ004	22c53(PL) 22c54(IM) 22c55(PCLT)	No	Yes	Yes	B	Yes	Yes	433	1,204.9	13.5	1.2	5
ZJ005	22c61(PL) 22c62(IM) 22c63(PCLT)	Yes	Yes	Yes	C	Yes	No	11,572	117,545.08	18.3	19.6	5

HBV, hepatitis B virus; MVI, microvascular invasion; BCLC, Barcelona clinic liver cancer; AFP, alpha fetoprotein; PIVKA-II, protein induced by vitamin K absence-II; CA199: carbohydrate antigen 199; CEA: carcinoembryonic antigen; RFS, relapse-free survival (months); PCLT, pericarcinomatous liver tissue; PL, primary lesion; IM, intrahepatic metastasis; IM-mHCC, intrahepatic metastatic multifocal hepatocellular carcinoma

Whole-exome sequencing (WES) was performed on matched tissue specimens to confirm the molecular pathology of these patient lesions as IM-mHCC. The results showed that most tumors carried mutations in genes such as *HMCN1* and *CSMD2* (Fig. 1C), consistent with the mutated genes in the Chinese Liver Cancer Atlas map of hepatitis B virus (HBV)-related HCC populations [18] (Fig. S1B). Moreover, the PL and IM in the same patient shared similar trunk mutations (Fig. 1C and D and Fig. S1C). Phylogenetic trees generated from somatic mutations to characterize IM [9] also revealed similar shared mutations between PL and IM in each patient, confirming them as IM-mHCC (Note: One patient was excluded due to nucleic acid degradation that failed to meet WES quality requirements) (Fig. 1D and Fig. S1C). Furthermore, a common copy number variation (CNV) was evident between the PL and IM groups (Fig. S1D). Further validation of IM-mHCC patient inclusion was performed using immunohistochemistry (IHC) staining, demonstrating lower CD20^+^ B cell intensity in the IM group compared with the MO group (Fig. S1E), a feature previously associated with metastatic dissemination [15]. Thus, we confirmed that the 5 included patients had IM-mHCC at various levels.

### Immune differences between PL and IM

**Fig. 2.**
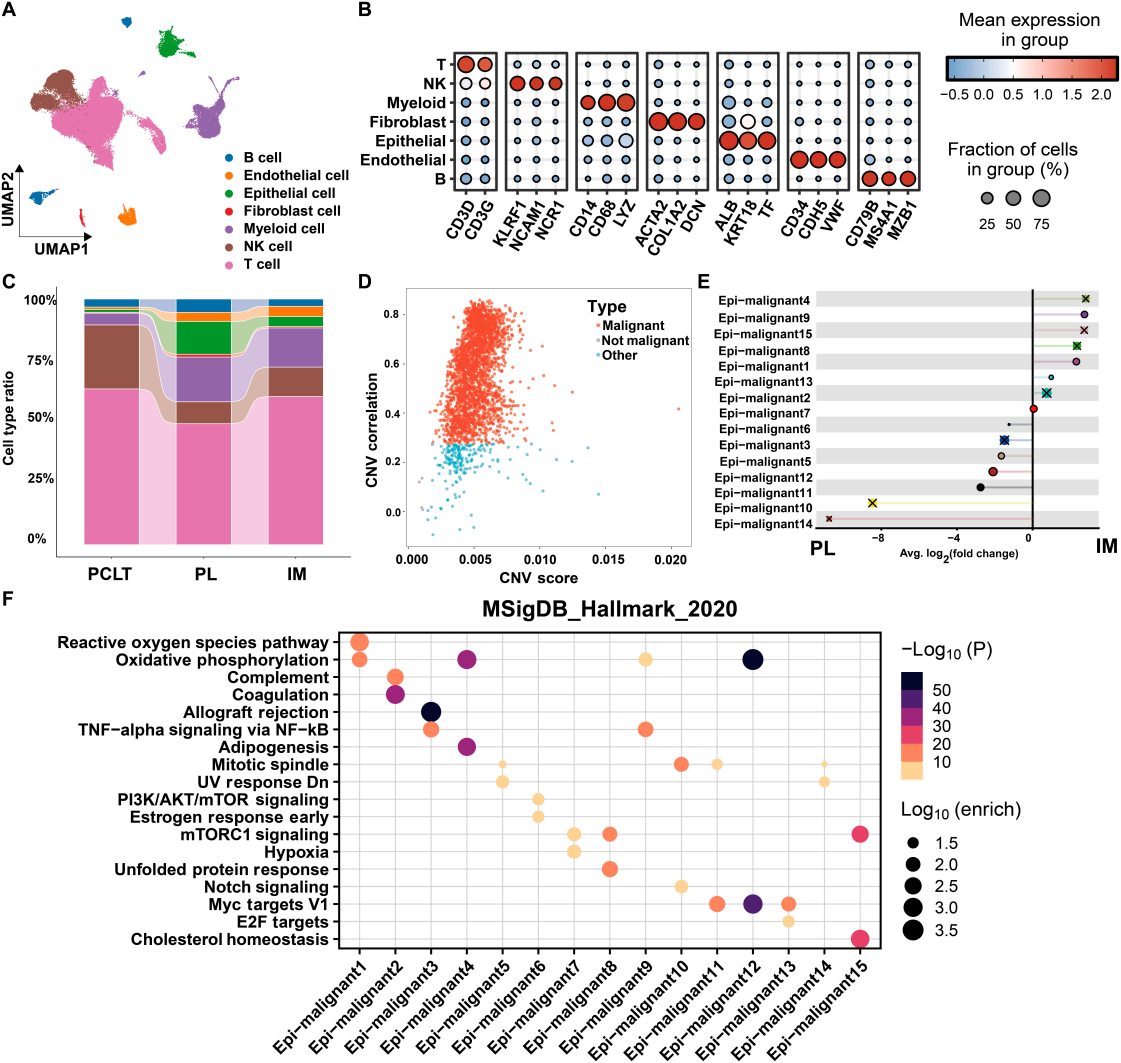
Single-cell transcriptomic analysis of intrahepatic metastatic multifocal hepatocellular carcinoma (IM-mHCC). (A) Uniform manifold approximation and projection (UMAP) plot displaying 7 major cell types across 15 samples from 5 patients with IM-mHCC. (B) Dot plot showing marker gene expression across major cell types. Color gradients reflect normalized expression magnitude, while dot diameter signifies the fraction of cells expressing each gene. (C) Stacked bar plot showing cellular composition proportions across the 3 tissue origins, colored by cell type. (D) Identification of malignant epithelial cells based on scatter plots of copy number variation (CNV) correlation coefficients and CNV scores relative to normal reference cells. (E) Lollipop plot of differential module eigengenes (intrahepatic metastasis [IM] vs. primary lesion [PL]) derived from high-dimensional weighted gene coexpression network analysis (hdWGCNA). Dot size corresponds to gene count per module; nonsignificant (ns) modules marked with “X.” (F) Bubble plots visualizing the top 2 enriched terms per module from the Molecular Signatures Database (MSigDB) Hallmark databases. Bubble size indicates the enrichment level, and color denotes significance. PCLT, pericarcinomatous liver tissue.

Utilizing cell-specific signature genes, we partitioned high-quality single cells into 7 fundamental lineages through dimensionality reduction techniques. These included lymphoid populations (T, NK natural killer, and B cells), myeloid cells, and stromal/structural components (fibroblasts, epithelial cells, and endothelial cells) (Fig. 2A and B and Fig. S2A). The identification of specific subtypes across these 7 major cell types was supported by functional enrichment analysis of cell type-specific up-regulated genes (Fig. S3). Next, we classified cells within the uniform manifold approximation and projection (UMAP) space into 3 populations based on their tissue origins, revealing diverse cellular ecosystems (Fig. S2B). Additionally, cells from different patients were thoroughly intermixed, ensuring that broad sampling did not introduce systematic biases (Fig. S2C). The IM group displayed increased T and NK cell proportions and decreased myeloid cell proportions compared with the PL group (Fig. 2C and Fig. S4A and B). We observed a shift from innate to adaptive immune cells during progression from PL to IM lesions. This suggests that during metastasis, tumors gradually adapt to the new environment, enabling tumor cells in IM to evade rapid immune clearance.

We applied the inferCNV algorithm to distinguish malignant from nonmalignant epithelial cells, which uncovered distinct CNV patterns in the PL, IM, and pericarcinomatous liver tissue (PCLT) (Fig. S4C). Using CNV-related heatmaps and CNV score scatter plots, malignant and nonmalignant epithelial cells were readily separated [19] (Fig. 2D). The CNV landscapes of malignant cells from matched PL and IM lesions exhibited high intrapatient concordance (Fig. S4D), which aligned with the WES results, further confirming that the included patients had IM-mHCC. UMAP visualization revealed patient-specific clustering of malignant cells influenced by their different origins (Fig. S4E), indicating substantial intertumoral heterogeneity.

To identify conserved transcriptomic alterations while mitigating patient-specific effects, we performed high-dimensional WGCNA on the malignant epithelial cells. This analysis partitioned the expression profiles into 15 distinct coexpression modules (Fig. S5A and B). Differential module eigengene analysis revealed that modules 5, 6, 11, and 12 were highly expressed in the PL, whereas modules 1, 9, and 13 were highly expressed in the IM (Fig. 2E and Fig. S5C and D). Kyoto Encyclopedia of Genes and Genomes (KEGG) and Molecular Signatures Database Hallmark pathway analyses (Fig. 2F and Fig. S5E), further corroborated by module hub gene network analysis (Fig. S6), revealed that oxidative phosphorylation, glycolysis, and fatty acid metabolism were activated in the IM group. These metabolic programs were driven by core hub genes such as COX7C and ENO1. Given that enhanced oxidative phosphorylation and fatty acid metabolism are crucial for supporting the energetic demands of tumor dissemination [20], their coordinated up-regulation highlights the aggressive metabolic landscape of IM-mHCC. Collectively, these findings suggest that malignant cells from different lesions have distinct characteristics, with those in the IM exhibiting stronger metabolic activity, proliferative capacity, and immunosuppressive features.

### Lymphoid cell subsets in IM-mHCC

**Fig. 3.**
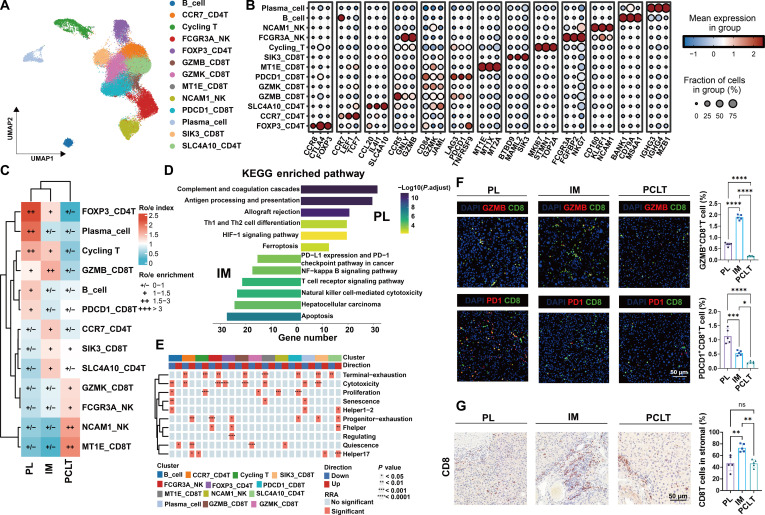
Single-cell resolution lymphoid cell atlas of intrahepatic metastatic multifocal hepatocellular carcinoma (IM-mHCC). (A) Uniform manifold approximation and projection (UMAP) visualization of lymphoid cell subsets. Colors represent different cell subsets. (B) Dot plot showing expression of marker genes in lymphoid cell subsets. (C) Ro/e (ratio of observed to expected cell numbers) indices for the different lymphoid cell subsets. Ro/e > 1 indicates higher-than-expected representation. (D) Kyoto Encyclopedia of Genes and Genomes (KEGG) pathway enrichment analysis (hypergeometric test, Benjamini–Hochberg-corrected *P* values) comparing T cell functions between primary lesion (PL) and intrahepatic metastasis (IM). (E) Heatmap of robust rank aggregation (RRA)-identified gene sets across the lymphoid cell subsets. Asterisks denote significance levels (top), and the dendrogram shows gene set similarity (left). The direction indicator (up or down) denotes whether the differential gene set is significantly enriched (up) or significantly depleted (down) in the specified cell cluster compared to all other clusters. (F) Representative multiplex immunohistochemistry images showing the distribution of GZMB^+^ CD8^+^ T cells and PD1^+^ CD8^+^ T cells in the PL, IM, and pericarcinomatous liver tissue (PCLT) of IM-mHCC. Scale bar, 50 μm. (G) Representative CD8 immunohistochemistry of PL, IM, and PCLTs. Five randomly selected regions were evaluated on each slide to enable comparative analysis of positive staining. Scale bar, 50 μm. ns, not significant.

After performing reclustering analysis of lymphoid cells and integrating specific marker genes, we identified 13 lymphoid cell subsets, including B cells, CCR7^+^ CD4^+^ T cells, cycling T cells, FCGR3A^+^ NK cells, FOXP3^+^ CD4^+^ T cells, GZMK^+^ CD8^+^ T cells, GZMB^+^ CD8^+^ T cells, MT1E^+^ CD8^+^ T cells, NCAM1^+^ NK cells, PDCD1^+^ CD8^+^ T cells, plasma cells, SIK3^+^ CD8^+^ T cells, and SLC4A10^+^ CD4^+^ T cells (Fig. 3A and B). We first examined the cell numbers and relative proportions of these subsets across lesions (Fig. S7A). Patient-level analysis revealed some variability in raw cellular proportions (Fig. S7B). To robustly determine the distribution preferences of lymphocytes among different lesions, we calculated the odds ratio (OR) and relative overexpectation (Ro/e) values. Among CD8^+^ T cells, GZMB^+^ CD8^+^ T cells were enriched in the IM, whereas PDCD1^+^ CD8^+^ T cells were enriched in the PL (Fig. 3C and Fig. S7C).

KEGG pathway enrichment analysis was conducted to explore the functional heterogeneity of T cells in the PL and IM groups. Compared with PCLTs, pathways enriched in tumor cells and associated with proliferation—including cell cycle and DNA replication—were found to be up-regulated (Fig. S7D). When comparing IM and PL, the IM group showed up-regulation of apoptosis, NF-kappa B signaling pathways, NK cell-mediated cytotoxicity, and programmed cell death 1 (PD-1)/programmed death-ligand 1 (PD-L1) pathways, while the PL group exhibited up-regulation of complement and coagulation cascades and antigen presentation pathways (Fig. 3D). Notably, the apoptosis and NF-kappa B signaling pathways are associated with T cell exhaustion, while PD-1/PD-L1 signaling is linked to immunosuppression [21,22]. This implies the presence of cells with high cytotoxic potential and exhaustion-related features in the IM.

We found that GZMB^+^ CD8^+^ T cells from the IM highly expressed markers related to interferon (IFN) activation, cytotoxicity, and exhaustion, whereas PDCD1^+^ CD8^+^ T cells from the PL highly expressed exhaustion- and tissue-resident memory-related markers (Fig. S8A). T cell state identifier (TCellSI) analysis indicated that GZMB^+^ CD8^+^ T cells highly expressed terminal exhaustion and cytotoxic-related functions, whereas PDCD1^+^ CD8^+^ T cells predominantly expressed exhaustion-related programs and proliferation (Fig. 3E). Transcription factor analysis also showed high expression of immune evasion-related transcriptional regulators, including MAF, BATF, STAT1, and IRF family members in these cells (Fig. S8B). Finally, we used multiplex immunohistochemistry (mIHC) to validate the expression of GZMB^+^ CD8^+^ T and PD-1^+^ CD8^+^ T cells in the PL, IM, and PCLTs. This analysis confirmed significant enrichment of GZMB^+^ CD8^+^ T cells in the IM and of PD-1^+^ CD8^+^ T cells in the PL (Fig. 3F). Spatial mapping revealed that CD8^+^ T cells in the IM were predominantly confined to tumor stromal regions with limited parenchymal infiltration (Fig. 3G). This compartmentalization suggests that despite cytotoxic potential, the impaired intratumoral trafficking in IM likely restricts CD8^+^ T cell effector functions and compromises tumor cell eradication.

To determine whether the GZMB^+^ CD8^+^ T cells enriched in the IM represent tumor-reactive and terminally exhausted clones, we performed multiplex immunofluorescence (mIF) staining for CD8, GZMB, CD39, and TCF1. A subset of CD8^+^CD39^+^TCF1^−^ cells was significantly enriched in the IM (Fig. S8C). Given that CD39 marks tumor-reactive CD8^+^ T cells and loss of TCF1 defines terminal exhaustion, these data suggest that the GZMB^+^ CD8^+^ T cells in the IM represent tumor-reactive clones that have progressed to terminal exhaustion.

Having established their terminal exhausted status in situ, we next sought to elucidate the developmental trajectory driving these T cells toward this fate. Pseudotime analysis with Monocle2 and Slingshot was conducted to resolve the developmental trajectory of CD8^+^ T cell subsets. The trajectory initiated from SIK3^+^ subsets and progressed toward terminally differentiated GZMB^+^ and PDCD1^+^ subsets (Fig. S9A and B). Kinetic analysis along the pseudotime identified 4 distinct gene clusters (C1 to C4). Although early stages (C1) were enriched in T cell activation signals, the terminal stage featured high expression of Clusters C3 and C4 (Fig. S9C). Specifically, Cluster C3 was enriched in genes associated with cytotoxicity (e.g., GZMA and CST7), while Cluster C4 was dominated by the unfolded protein response and negative regulation of the mitogen-activated protein kinase cascade (e.g., DUSP1 and NFKBIA). This coexpression pattern indicates that terminally differentiated GZMB^+^ CD8^+^ T cells in the IM experience substantial proteostatic stress and active suppression of TCR signaling, both of which are hallmark features of functional exhaustion. This terminal state was further validated by Slingshot analysis, which positioned GZMB^+^ CD8^+^ T cells at the end of lineage 1 (Fig. S9D). Collectively, these findings confirm the presence of an immunosuppressive TIME in intrahepatic metastases, contributing to tumor immune evasion.

### GPNMB^+^ macrophages in IM and CD8^+^ T cell interactions

**Fig. 4.**
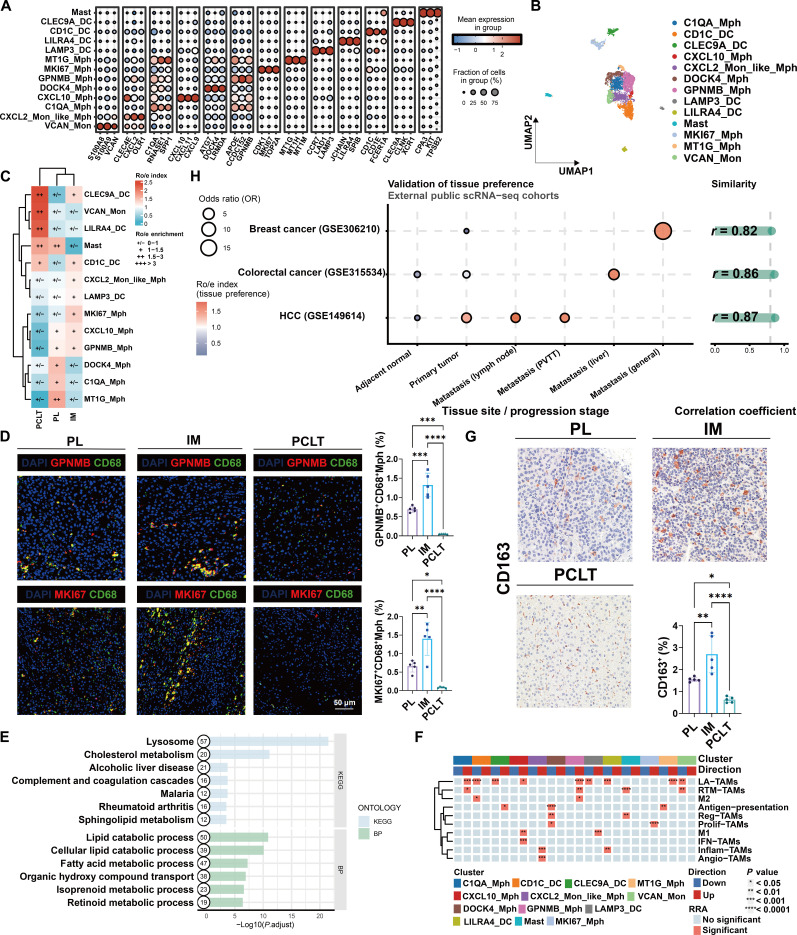
Characterization of myeloid cells in intrahepatic metastatic multifocal hepatocellular carcinoma (IM-mHCC). (A) Dot plot showing expression of marker genes in myeloid cell subsets. (B) Uniform manifold approximation and projection (UMAP) plot of myeloid cell subsets colored according to the different cell subsets. (C) Ro/e (ratio of observed to expected cell numbers) indices for different myeloid cell subsets. Ro/e > 1 indicates higher-than-expected representation. (D) Representative multiplex immunohistochemical (mIHC) staining images indicating glycoprotein nonmetastatic melanoma protein B-positive (GPNMB^+^) macrophages (Mph) and MKI67^+^ Mph in the primary lesion (PL), intrahepatic metastasis (IM), and pericarcinomatous liver tissue (PCLT). Scale bar, 50 μm. (E) Gene Ontology (GO) and Kyoto Encyclopedia of Genes and Genomes (KEGG) enrichment analysis of marker genes in GPNMB^+^ Mph. (F) Heatmap of robust rank aggregation (RRA)-identified gene sets across the myeloid subsets. Asterisks denote significance levels (top), and the dendrogram shows gene set similarity (left). The direction indicator (up or down) denotes whether the differential gene set is significantly enriched (up) or significantly depleted (down) in the specified cell cluster compared to all other clusters. (G) Representative images of CD163 immunohistochemistry of PL, IM, and PCLTs. Scale bar, 50 μm. (H) Bubble plot showing the odds ratio (OR) and Ro/e of GPNMB^+^ macrophages across adjacent normal, primary tumor, and diverse metastatic sites in breast cancer, colorectal cancer, and hepatocellular carcinoma single-cell RNA sequencing (scRNA-seq) datasets. The right panel displays the cross-cohort transcriptional correlation coefficients (*r* > 0.8) for GPNMB^+^ macrophages. ns, not significant.

Based on specific marker genes, we identified 13 myeloid cell subsets. The macrophages were designated as C1QA^+^ Mph, CXCL10^+^ Mph, CXCL2^+^ Mph, DOCK4^+^ Mph, GPNMB^+^ Mph, MKI67^+^ Mph, and MT1G^+^ Mph (Fig. 4A and B). We first examined their relative proportions across lesion types (Fig. S10A). Patient-level analysis also revealed some variability in raw cellular percentages (Fig. S10B). Subsequent analyses revealed that GPNMB^+^ Mph and MKI67^+^ Mph exhibited the highest OR and Ro/e within macrophages in the IM, whereas C1QA^+^ Mph and DOCK4^+^ Mph were predominant in the PL (Fig. 4C and Fig. S10C). Furthermore, we validated the spatial distribution differences of these 4 types of macrophages between the PL and IM groups using mIHC (Fig. 4D).

To characterize the heterogeneity of monocytes/macrophages across lesions, Gene Ontology and KEGG pathway enrichment analyses were performed. The IM group was significantly enriched in aerobic respiration, oxidative phosphorylation, and chromosome segregation, suggesting that the metastatic microenvironment demands higher metabolic activity and promotes self-renewal. The PL group exhibited up-regulation of complement and coagulation cascades and cell–substrate adhesion pathways, implying involvement in tumor progression through facilitation of metastasis [23,24] (Fig. S10D). GPNMB^+^ Mph enriched in IM exhibited up-regulated cholesterol metabolism and lipid catabolism pathways, suggesting their role in promoting immune evasion through lipid metabolic reprogramming (Fig. 4E). These metabolic features coexisted with an immunosuppressive TIME in the IM [25,26]. Furthermore, classification using established macrophage polarization markers demonstrated that GPNMB^+^ Mph represented lipid-associated tumor-associated macrophages, whereas MKI67^+^ Mph corresponded to a proliferative subset [27] (Fig. 4F). Consistent with their immunosuppressive activity, GPNMB^+^ macrophages expressed higher levels of M2-associated genes compared to other TAMs (Fig. 4F and Fig. S10E). Immunohistochemical validation confirmed elevated CD163^+^ cell infiltration in IM relative to PL and PCLTs (Fig. 4G).

To determine whether the metastasis-driven enrichment of this specific immunosuppressive population extends beyond our IM-mHCC cohort, we analyzed independent public scRNA-seq metastasis cohorts across HCC with portal vein tumor thrombus, colorectal cancer, and breast cancer. Consistent with our cohort, GPNMB^+^ macrophages showed a strong preference for metastatic niches (e.g., portal vein tumor thrombus and liver metastasis), evidenced by significantly elevated OR and Ro/e (Fig. 4H and Fig. S11). Additionally, these macrophage populations exhibited high cross-cancer transcriptional similarity (*r* > 0.8) (Fig. 4H). These external validations robustly confirm the widespread enrichment of GPNMB^+^ macrophages within metastatic microenvironments.

To resolve the developmental ontogeny and transition of monocytes/macrophages subsets, we performed trajectory analysis using Slingshot and sctour. Slingshot analysis delineated 4 distinct lineages and positioned GPNMB^+^ Mph at the intermediate-to-late stage of Lineage 1 (Fig. S12A and B). Consistently, sctour vector field analysis revealed a directional transcriptomic flow originating from MKI67^+^ Mph and VCAN^+^ Mon toward mature macrophage states (Fig. S12C to E). Density distribution along the pseudotime axis further confirmed that GPNMB^+^ Mph were predominantly concentrated at the intermediate-to-late stage of the developmental spectrum (Fig. S12F). Collectively, these findings suggest that GPNMB^+^ Mph represent a niche-adapted and functionally mature terminal subpopulation within the IM.

**Fig. 5.**
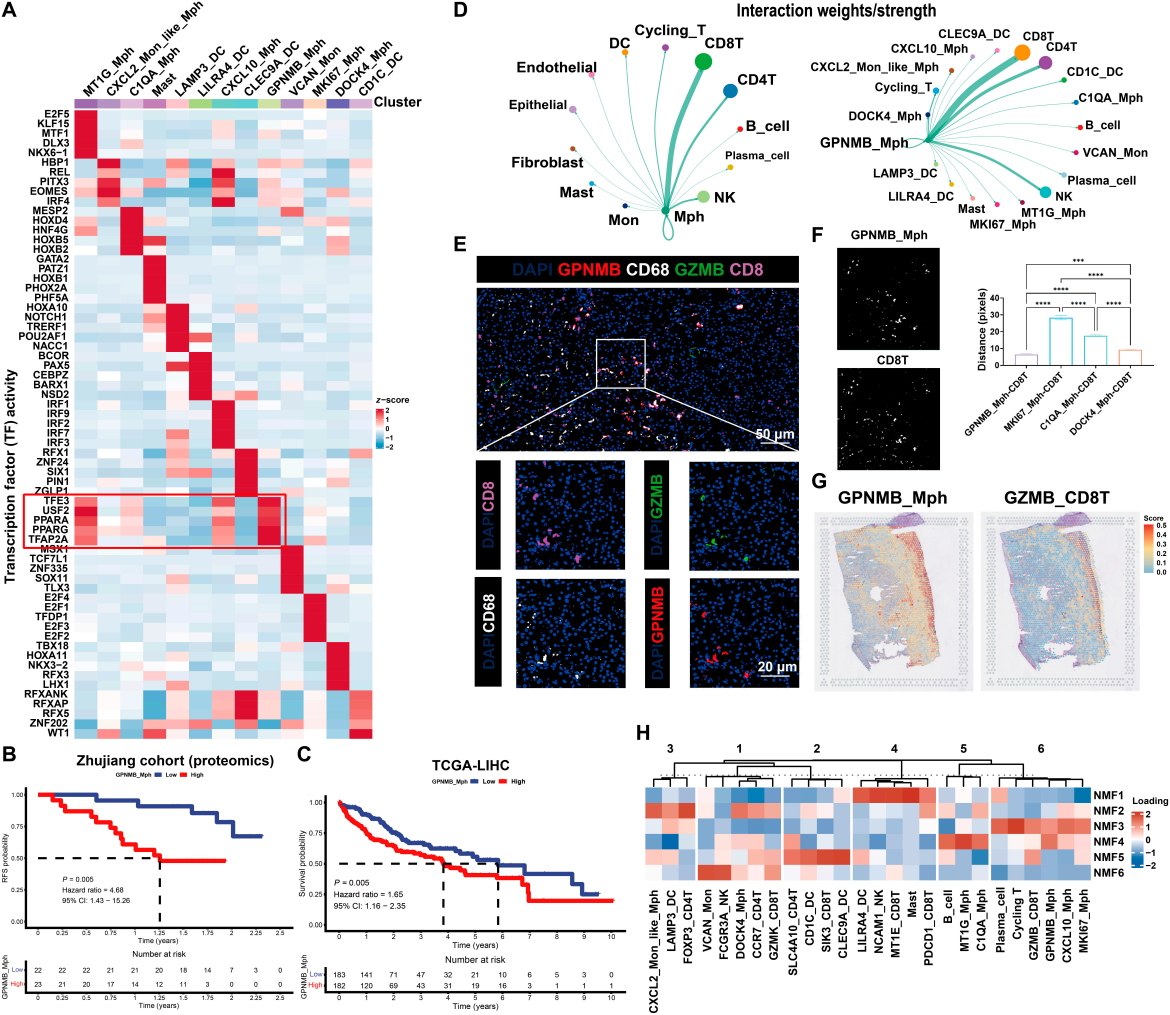
Characterization of GPNMB^+^ macrophages (Mph) in intrahepatic metastatic multifocal hepatocellular carcinoma (IM-mHCC). (A) Heatmap mapping transcription factor regulon activity across the identified myeloid cell subsets. (B) Kaplan–Meier survival analysis of recurrence-free survival (RFS) in the Zhujiang Proteomics Cohort, stratified by high versus low GPNMB^+^ Mph scores; log-rank testing was used for statistical evaluation (C) Kaplan–Meier survival analysis of overall survival (OS) in the Cancer Genome Atlas Liver Hepatocellular Carcinoma (TCGA-LIHC) dataset stratified by high versus low GPNMB^+^ Mph scores; log-rank testing was used for statistical evaluation. (D) Circle plot visualizing the interaction strength between major cell categories (left chart) and the interaction strength between myeloid cell subsets and lymphoid cells (right chart). (E) Representative regions depicting cellular niches, including GPNMB^+^ Mph and GZMB^+^ CD8^+^ T cells, across the intrahepatic metastasis (IM), using multiplex immunohistochemistry (mIHC). Scale bars, 50 μm (main overview) and 20 μm (magnified insets). (F) The distance between macrophage subsets (GPNMB^+^ Mph, MKI67^+^ Mph, DOCK4^+^ Mph, and C1QA^+^ Mph) and CD8^+^ T cells. Multiplex immunohistochemical localization images of GPNMB^+^ Mph and CD8^+^ T cells, with white spots indicating cell positions (left). (G) Spatial transcriptomics analysis of colocalization between GPNMB^+^ Mph and GZMB^+^ CD8^+^ T cells. (H) Tumor microenvironment (TME) cellular programs derived from non-negative matrix factorization (NMF). Cell types within the TME are displayed on the abscissa, whereas the ordinate indicates the factor loading values of each cell type across distinct programs. PCLT, pericarcinomatous liver tissue; PL, primary lesion. ***P < 0.001, ****P < 0.0001.

Transcription factor profiling identified peroxisome proliferator-activated receptor alpha (PPARα), upstream stimulatory factor 2 (USF2), nuclear receptor subfamily 1 group H member 3 (NR1H3), and zinc finger protein of the cerebellum 2 (ZIC2) as highly expressed in GPNMB^+^ Mph (Fig. 5A). Specifically, PPARα and NR1H3 regulate cholesterol/lipid metabolism, whereas USF2 and ZIC2 may promote tumor proliferation and metastatic invasion [28,29]. To evaluate the clinical impact of these protumorigenic signatures, we analyzed independent patient cohorts. In the Zhujiang Proteomics Cohort, high GPNMB^+^ macrophage infiltration correlated with significantly worse recurrence-free survival (*P* = 0.005, hazard ratio = 4.68) (Fig. 5B), corroborating the reduced overall survival observed in the The Cancer Genome Atlas Liver Hepatocellular Carcinoma dataset (Fig. 5C). Furthermore, an independent triple-negative breast cancer scRNA-seq cohort revealed that GPNMB^+^ TAMs were enriched in patients with stable disease versus partial response (Fig. S13A and B), implicating this subset in immunotherapy resistance.

Integrating untargeted metabolomics from the Zhujiang cohort, GPNMB-high samples exhibited distinct alterations in lipid metabolites (Fig. S13C). Pathway enrichment highlighted lipid and cholesterol homeostasis, notably ATP-binding cassette transporters and bile secretion (Fig. S13D). This orthogonally validates the upstream activation of lipid-associated transcription factors (PPARα/NR1H3) identified in our single-cell analysis. Concurrently, immunosuppressive circuits, particularly tryptophan metabolism, were up-regulated. Together, these multiomics data demonstrate that the poor clinical outcomes associated with GPNMB^+^ macrophages are driven by coordinated lipid metabolic rewiring and microenvironmental immunosuppression.

To investigate the potential communication mechanisms among macrophage subsets, we performed cell–cell interaction analysis. The findings indicated a substantial interaction between the macrophages and CD8^+^ T cells (Fig. 5D, left). Focusing specifically on GPNMB^+^ Mph and lymphocyte subsets, the interaction analysis demonstrated strong interactions between GPNMB^+^ Mph and CD8^+^ T cells, particularly between PDCD1^+^ CD8^+^ T cells and GZMB^+^ CD8^+^ T cells, suggesting potential regulatory networks (Fig. 5D, right and Fig. S14A). This spatial association was validated by mIF, which showed close proximity between GPNMB^+^ Mph/MKI67^+^ Mph and GZMB^+^ CD8^+^ T cells in the IM and between DOCK4^+^ Mph/C1QA^+^ Mph and PDCD1^+^ CD8^+^ T cells in the PL (Fig. 5E and Fig. S14B). Quantitative spatial distance analysis of these 4 macrophage subsets and CD8^+^ T cells confirmed that GPNMB^+^ Mph exhibited the shortest physical distance to CD8^+^ T cells, supporting their strong interaction (Fig. 5F). Spatial transcriptomic analysis further revealed the significant colocalization of GPNMB^+^ Mph and GZMB^+^ CD8^+^ T cells (Fig. 5G and Fig. S14C). Further exploration of specific myeloid–lymphoid interaction pathways revealed that GPNMB^+^ Mph primarily engages other cells via the MHC-I, CXCL, GALECTIN, and SPP1 signaling pathways (Fig. S15A). Notably, the SPP1 pathway has been shown to play a role in mediating the immunosuppressive TIME [30,31]. Comparative communication analysis of GPNMB^+^ Mph and CD8^+^ T cell subsets between the PL and IM showed enhanced interactions between GPNMB^+^ Mph and GZMB^+^ CD8^+^ T cells in the IM, along with significant activation of the PTGES3–PTGER4, LIPA–RORA, and HLA–CD8 signaling pathways (Fig. S15B). To further validate these inferred interactions, we performed mIF staining to evaluate the spatial coexpression of these 3 prioritized ligand-receptor pairs. In all 3 panels, GPNMB^+^ macrophages were observed in close proximity to CD8^+^ T cells within the IM, with clear coexpression of the respective ligand and receptor molecules (Fig. S16). These spatial data provide histological support for the CellChat-predicted crosstalk between GPNMB^+^ macrophages and CD8^+^ T cells.

Finally, non-negative matrix factorization (NMF) analysis was applied to identify coordinated cellular programs within the TIME. This approach identified 6 distinct metaprograms (NMF1 to NMF6). Notably, GPNMB^+^ Mph, MKI67^+^ Mph and GZMB^+^ CD8^+^ T cells from the IM were coenriched in NMF3 (metabolism-associated metaprogram) (Fig. 5H). This finding further validates their functional interactions and suggests a potential crosstalk via metabolic pathways.

### GPNMB^+^ macrophages suppress CD8^+^ T cell cytotoxicity

Previous studies have indicated the immunosuppressive functions of GPNMB in glioma and colorectal cancer [32,33]. Given the immunosuppressive signature of GPNMB^+^ macrophages [34], we further investigated their role in shaping the immunosuppressive TIME of IM. First, we established macrophage models with GPNMB overexpression in THP1 and RAW264.7 cells (Fig. S17A). Transwell migration and scratch wound healing assays demonstrated that GPNMB^+^ Mph significantly enhanced tumor cell migration compared to controls, confirming their direct protumorigenic function (Fig. S17B and C).

**Fig. 6.**
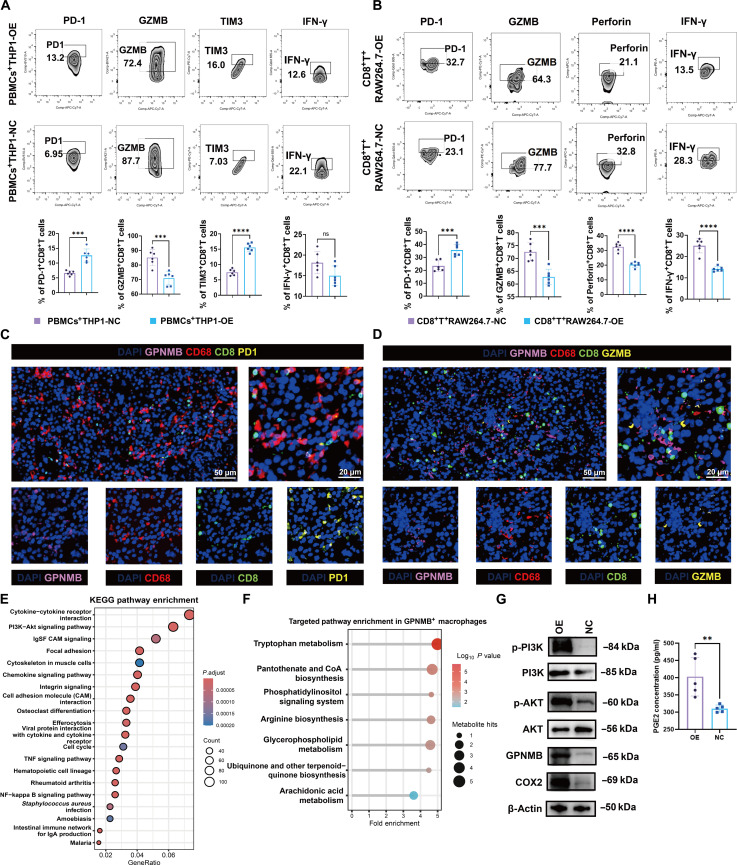
In vitro validation of the protumorigenic role of GPNMB^+^ macrophages. (A) Flow cytometry analysis of PD-1, GZMB, TIM3, and interferon-γ (IFN-γ) expression in human CD8^+^ T cells cocultured with GPNMB^+^ macrophages. (B) Flow cytometry analysis of PD-1, GZMB, Perforin, and IFN-γ expression in murine CD8^+^ T cells cocultured with GPNMB^+^ macrophages. (C) Spatial relationship verification among GPNMB^+^ macrophages, CD8^+^ T cells, and PD-1 molecules via multiplex immunofluorescence (mIF). Scale bars, 50 μm (main overview) and 20 μm (magnified insets). DAPI, 4′,6-diamidino-2-phenylindole. (D) Spatial relationship verification among GPNMB^+^ macrophages, CD8^+^ T cells, and GZMB molecules via mIF. Scale bars, 50 μm (main overview) and 20 μm (magnified insets). (E) Kyoto Encyclopedia of Genes and Genomes (KEGG) pathway enrichment analysis of transcriptomic profiles from GPNMB-overexpressing macrophages. (F) Targeted pathway enrichment analysis based on the untargeted metabolomics profiling of GPNMB-overexpressing macrophages. (G) Western blot analysis of phosphorylated phosphatidylinositol 3-kinase (p-PI3K), PI3K, p-AKT, AKT, GPNMB, and cyclooxygenase-2 (COX2) in negative control (NC) and GPNMB-overexpressing (OE) macrophages. β-actin was used as the loading control. (H) Enzyme-linked immunosorbent assay quantification of prostaglandin E2 (PGE2) secretion in the culture supernatants of NC and OE macrophages. Data are presented as means ± SEM. PBMCs, peripheral blood mononuclear cells; ns, not significant. **P < 0.01, ***P < 0.001, ****P < 0.0001.

To delineate the regulatory effect of GPNMB^+^ Mph on CD8^+^ T cell functionality, we performed coculture experiments using human and murine macrophages with their homologous CD8^+^ T cells respectively, followed by flow cytometry. GPNMB^+^ Mph suppressed the expression of cytotoxic effector molecules (GZMB, IFN-γ, and perforin) in CD8^+^ T cells, impairing their tumor-killing capacity. Concurrently, they up-regulated immunosuppressive checkpoints (PD-1 and TIM3) in CD8^+^ T cells, thereby establishing an immunosuppressive niche (Fig. 6A and B). Analysis of clinical intrahepatic metastatic specimens by mIF revealed significant colocalization of GPNMB^+^ Mph and CD8^+^ T cells, suggesting direct cellular crosstalk (Fig. 6C and D). Critically, PD-1 was enriched in proximity to GPNMB^+^ Mph, whereas GZMB was sparsely expressed. This spatial pattern indicates that GPNMB^+^ Mph may orchestrate immunosuppression by inducing PD-1 expression and suppressing GZMB expression in neighboring T cells.

Guided by our cell communication analysis implicating prostaglandin E2 (PGE2)-mediated crosstalk between GPNMB^+^ macrophages and CD8^+^ T cells, we investigated the intracellular mechanisms driving PGE2 synthesis. Transcriptomic and metabolomic profiling of GPNMB-overexpressing macrophages revealed significant enrichment of the phosphatidylinositol 3-kinase (PI3K)-Akt pathway (Fig. 6E) and a concurrent shift toward glycerophospholipid and arachidonic acid metabolism, the direct precursors of prostaglandins (Fig. 6F). Mechanistically, immunoblotting confirmed that GPNMB overexpression increased PI3K and AKT phosphorylation while up-regulating COX2, the rate-limiting enzyme in PGE2 synthesis (Fig. 6G). Accordingly, enzyme-linked immunosorbent assay confirmed significantly elevated PGE2 secretion (Fig. 6H). Together, these results demonstrate that GPNMB^+^ macrophages enforce an immunosuppressive niche via PI3K/AKT-COX2-driven PGE2 signaling.

### APL_siGpnmb_ targets Gpnmb^+^ macrophages in vivo

**Fig. 7.**
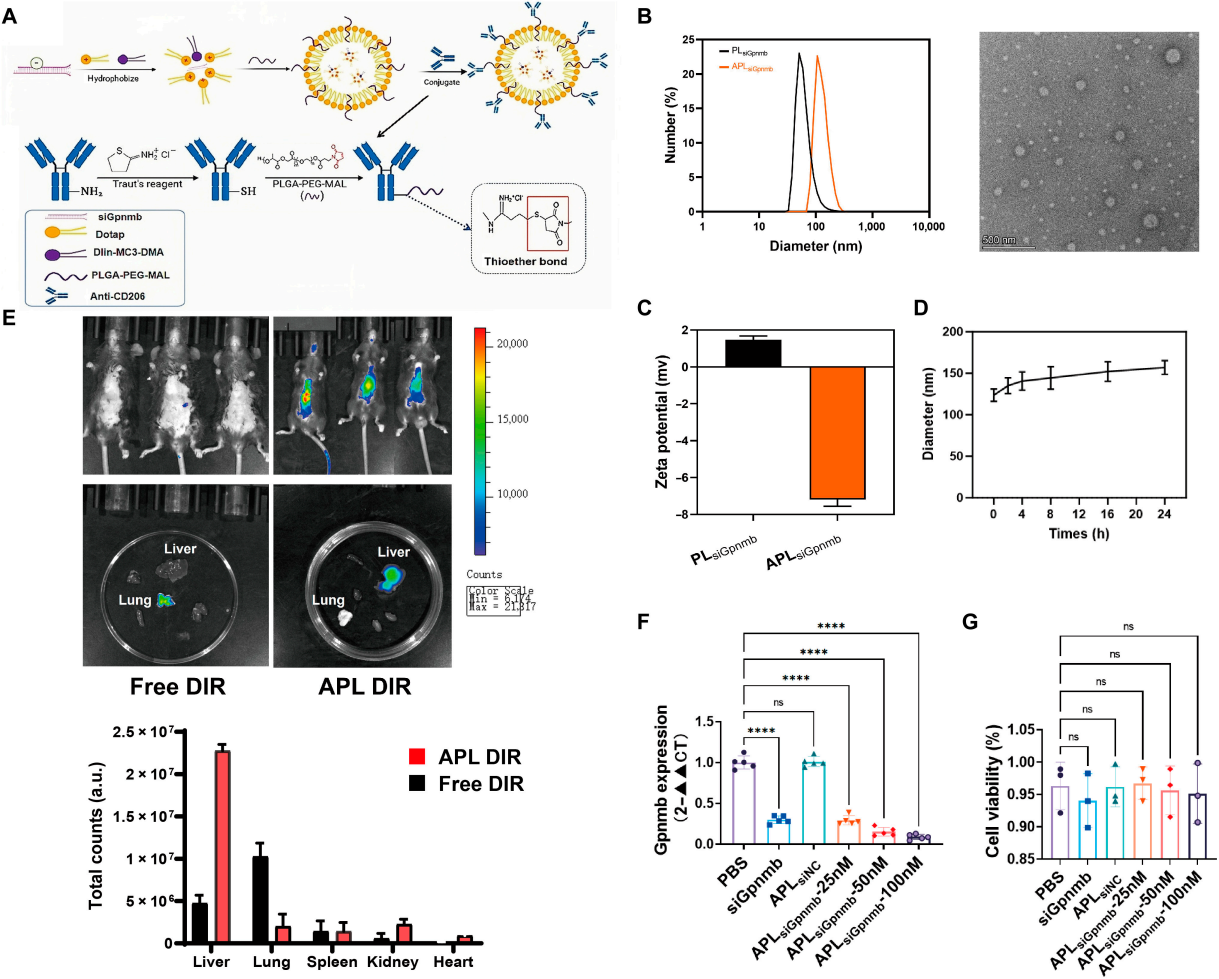
Preparation and characterization of CD206 antibody-modified lipid nanoparticles encapsulating siGPNMB for macrophage targeting (APL_siGpnmb_). (A) APL_siGpnmb_ synthesis schematic diagram. (B) The size distributions of APL_siGpnmb_ and plain lipid nanoparticles without antibody modification (PL_siGpnmb_) that belonged to 3 different batches and transmission electron microscopy (TEM) images of APL_siGpnmb_. Scale bar: 500 nm. (C) Zeta potential of APL_siGpnmb_ and PL_siGpnmb_. (D) Particle stability of APL_siGpnmb_ in phosphate-buffered saline (PBS). The particle size measured at each time period is marked in the figure. (E) In vivo and ex vivo imaging of major organs and tumor tissues was performed 24 h after APL-DIR (1,1'-dioctadecyl-3,3,3',3'-tetramethylindotricarbocyanine iodide) injection and quantification of DIR fluorescence signals. (F) Quantitative real-time polymerase chain reaction (qRT-PCR) analysis of siGpnmb expression in RAW264.7 cells after various treatments. (G) RAW264.7 cells were treated with APL_siGpnmb_ at various concentrations, and then the cell viability was assessed. CCK-8, Cell Counting Kit-8; ns, not significant. ****P < 0.0001.

To validate the role of Gpnmb^+^ Mph in vivo, we developed an antibody-modified polymer–lipid composite nanocarrier (APL_siGpnmb_) designed to encapsulate Gpnmb-specific small interfering RNA (siGpnmb). The nanoparticle surface was functionalized with CD206 antibodies to ensure precise targeting of tumor-associated macrophages (Fig. 7A). Characterization of APL_siGpnmb_ nanoparticles revealed a particle size of approximately 100 nm, facilitating efficient tumor penetration (Fig. 7B). Its monodisperse distribution indicated a stable preparation process, preventing rapid clearance. The uniform spherical morphology promoted cellular uptake via endocytosis, while the absence of aggregation reduced the risk of embolism or inflammation upon intravenous injection. The shift from positive to negative zeta potential after antibody conjugation prolonged circulation time, enhancing tumor accumulation (Fig. 7C). Furthermore, APL_siGpnmb_ exhibited favorable serum stability (Fig. 7D). We also validated the liver-targeting specificity of APL_siGpnmb_ over other organs in mouse HCC models (Fig. 7E). Quantitative real-time polymerase chain reaction results confirmed that APL_siGpnmb_ effectively silenced Gpnmb mRNA expression in macrophages, and the silencing efficacy was dose-dependent (Fig. 7F). Cell Counting Kit-8 assay confirmed its favorable biosafety profile (Fig. 7G).

**Fig. 8.**
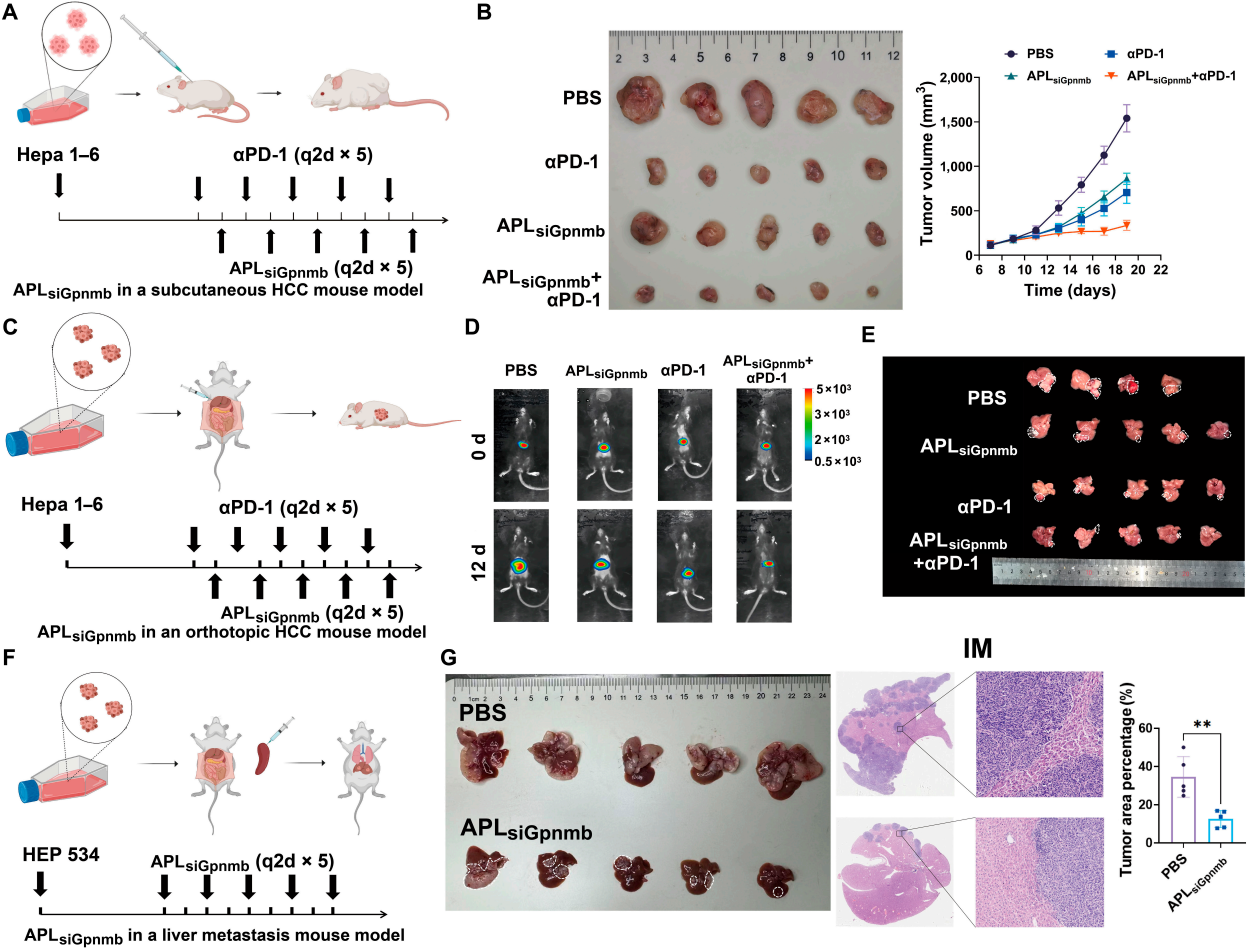
Validation of the therapeutic efficacy of CD206 antibody-modified lipid nanoparticles encapsulating siGpnmb for macrophage targeting (APL_siGpnmb_) in various mouse models. (A) Schematic diagram of APL_siGpnmb_ treatment in a mouse subcutaneous tumor model. (B) Representative ex vivo tumor specimens (left) and tumor growth curves (right) from the phosphate-buffered saline (PBS) control, anti-programmed death-1 (anti-PD-1) monotherapy, APL_siGpnmb_ monotherapy, and APL_siGpnmb_ + anti-PD-1 combination groups. (C) Schematic diagram of APL_siGpnmb_ treatment in a mouse orthotopic tumor model. (D) Small-animal in vivo bioluminescence imaging evaluating tumor burden across the 4 treatment groups. (E) Representative ex vivo tumor specimens from orthotopic tumor-bearing mice. (F) Schematic diagram of APL_siGpnmb_ treatment in a splenic injection model of liver metastasis. (G) Representative ex vivo tumor specimens (left) and hematoxylin and eosin (H&E)-stained images of intrahepatic nodules (right) from the PBS control, and APL_siGpnmb_ monotherapy groups. IM, intrahepatic metastasis. **P < 0.01.

**Fig. 9.**
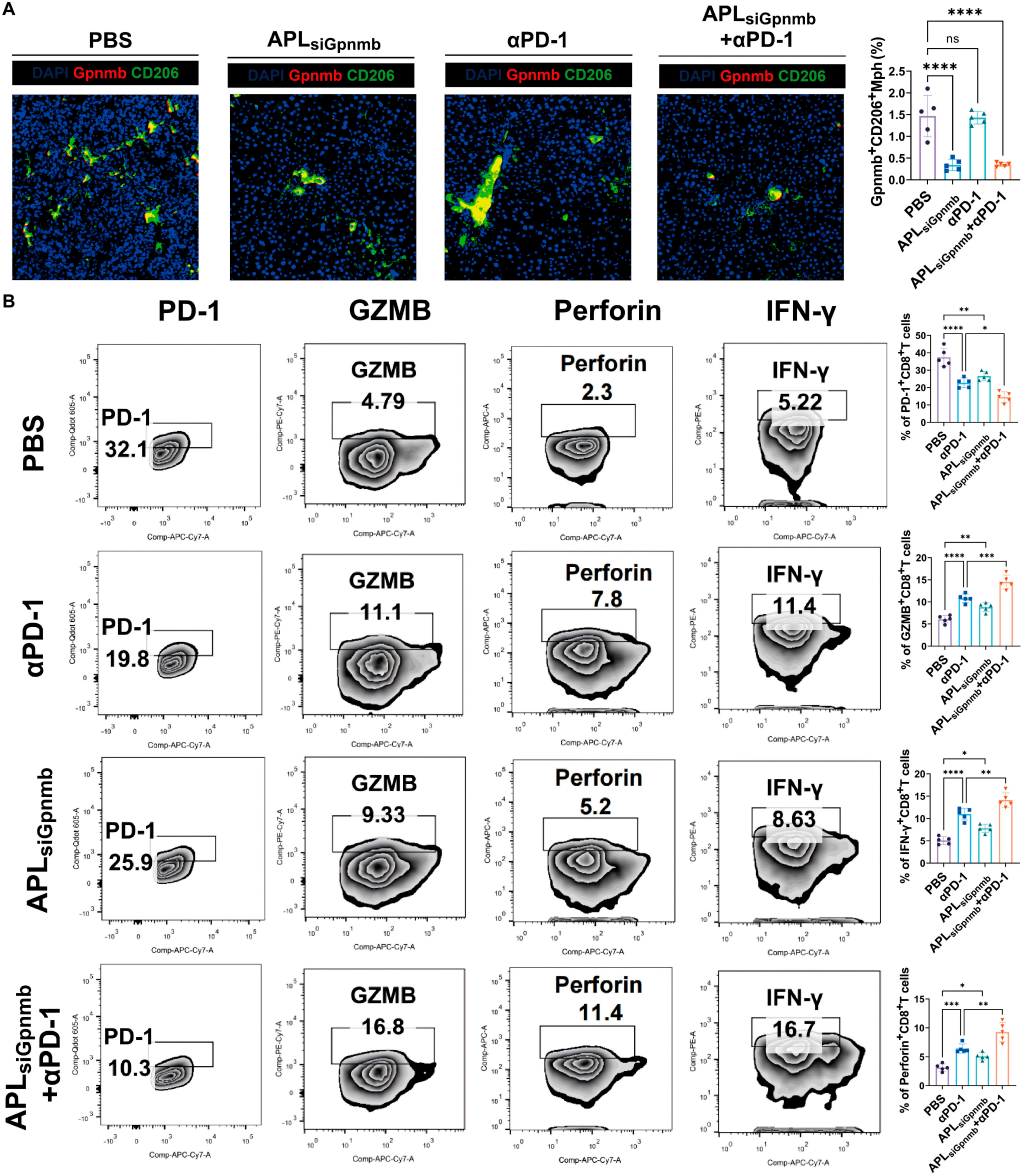
In vivo validation of the effect of CD206 antibody-modified lipid nanoparticles encapsulating siGpnmb for macrophage targeting (APL_siGpnmb_). (A) Multiplex immunohistochemistry validation of Gpnmb^+^ macrophage expression in mouse models treated with APL_siGpnmb_. DAPI, 4′,6-diamidino-2-phenylindole. (B) Flow cytometry analysis of programmed death-1 (PD-1), GZMB, Perforin, and interferon-γ (IFN-γ) expression levels in CD8^+^ T cells across 4 treatment groups. PBS, phosphate-buffered saline; mIHC, multiplex immunohistochemistry. *P < 0.05, **P < 0.01, ***P < 0.001, ****P < 0.0001.

Next, we evaluated the therapeutic efficacy of APL_siGpnmb_ in orthotopic, subcutaneous, and liver metastasis models. In C57BL/6 mice bearing subcutaneous tumors, both APL_siGpnmb_ and anti-PD-1 monotherapies significantly suppressed tumor growth relative to controls, with combination therapy demonstrating synergistic efficacy (Fig. 8A and B). Comparable tumor inhibition was observed in the orthotopic liver tumors (Fig. 8C to E and Fig. S18A). To better model IM-mHCC, a mouse HCC model with hepatic metastasis was established by splenic vein injection. APL_siGpnmb_ group showed a marked decrease in both tumor number and size compared with the phosphate-buffered saline group, as confirmed by both macroscopic examination and histopathological analysis with hematoxylin and eosin staining (Fig. 8F and G). mIHC confirmed reduced Gpnmb^+^ Mph infiltration in the APL_siGpnmb_ group, verifying in vivo target engagement (Fig. 9A). Flow cytometry analysis found that APL_siGpnmb_ and/or anti-PD-1 treatment enhanced CD8^+^ T cell cytotoxic function (elevated GZMB/IFN-γ/perforin) and reversed T cell exhaustion (Fig. 9B). Critically, APL_siGpnmb_ administration resulted in no adverse effects on body weight, hematopoiesis, or hepatic/renal function (ALT, AST, BUN, and creatinine) (Fig. S18B to D). To rigorously assess potential delayed toxicity and systemic immune perturbation, we further performed a 1-month longitudinal follow-up in healthy, immunocompetent mice. Consistent with the short-term data, extended APL_siGpnmb_ administration induced no histopathological lesions across major organs (Fig. S18E). Most notably, splenic flow cytometry confirmed that systemic immune homeostasis was strictly preserved, with unchanged frequencies of major lymphoid (CD4^+^/CD8^+^ T, and NK) and myeloid subsets (Fig. S18F). These combined results validate the excellent in vivo biocompatibility and translational safety of the APL_siGpnmb_ system.

## Discussion

Despite significant advancements in the treatment of HCC, patients with IM-mHCC continue to have a poor prognosis, reflected in a 5-year postoperative survival rate of only 20% to 30% [35]. Here, by profiling PL, IM, and PCLTs at single-cell resolution, we delineate lesion-specific features of the TIME. We identified a macrophage-centered, therapeutically actionable axis that explains the clinical aggressiveness of IM-mHCC. Specifically, we identify and validate that GPNMB^+^ Mph serve as a key immunosuppressive cell subset in the IM of IM-mHCC, orchestrating T cell dysfunction and promoting tumor progression. Furthermore, we engineered APL_siGpnmb_ nanoparticles capable of specifically silencing GPNMB^+^ Mph, which reversed CD8^+^ T cell dysfunction and reshaped the TIME.

Through integration of pathological and biological tumor characteristics, genomic analysis, and single-cell CNV analysis, we confirmed that PL and IM in IM-mHCC share similar pathological features, gene mutations, and CNV patterns. Additionally, the IM showed markedly fewer B cells than the PL, pointing to a shift in immune composition [15]. We also found that the malignant cells isolated from the IM exhibited higher metabolic activity, proliferative capacity, and immunosuppressive features than those isolated from the PL. In contrast, malignant cells from the PL activated inflammation-related and apoptotic pathways, illustrating the heterogeneity between PL and IM. These findings emphasize the need to develop personalized treatment approaches based on the distinct characteristics of different lesions.

In HCC, tumor-induced T cell exhaustion plays a pivotal role in both disease progression and resistance to immunotherapy [36]. This study evaluated differences in spatial distribution, heterogeneity, and functional attributes of immune cell subtypes between PL and IM. Compared to CD8^+^ T cell subsets in the PL, IM exhibited a significant enrichment of GZMB^+^ CD8^+^ T cells, which coexpressed markers and transcriptional signatures associated with both cytotoxic potential and T cell exhaustion. This resembles the dysfunctional CD8^+^ T cell subsets reported in microvascular invasion-positive HCC [37]. Similar to terminally exhausted T cells (TEX-term), GZMB^+^ CD8^+^ T cells exhibit heightened cytotoxic potential but functionally represent terminal exhaustion, characterized by shortened lifespans and loss of self-renewal capacity that fails to control tumor growth [38]. Moreover, through IHC localization, we found that CD8^+^ T cells were mainly enriched in the tumor stroma rather than in the tumor interior, indicating that an immune barrier prevents CD8^+^ T cells from infiltrating and exerting their killing function within the tumor. When effector immune cells are present in the tumor stroma, even if they possess cytotoxic killing functions, they cannot exert cytotoxic effects within the tumor, leading to tumor immune escape [39]. These findings demonstrate that GZMB^+^ CD8^+^ T cells possess both cytotoxic and exhausted features in the IM, which may explain the clinical phenomenon of tumor drug resistance or recurrence of IM.

Functional heterogeneity of macrophages in the TIME significantly affects HCC immune regulation and clinical outcomes. Recent scRNA-seq studies highlighted the crucial role of macrophages in the TIME regulation [40,41]. Our research identified GPNMB^+^ Mph and MKI67^+^ Mph as enriched in the IM, which activate lipid metabolism and cell proliferation pathways, respectively. Compared with previous studies identifying diverse TAM subsets in primary HCC, such as C1QA^+^ or SPP1^+^ macrophages, our findings highlight GPNMB^+^ Mph as a distinct, metastasis-enriched population. While conventional TAMs exhibit widespread distribution across tumors, GPNMB^+^ Mph demonstrate a unique spatial preference for the IM niche, indicating specific adaptation to the metastatic microenvironment. To our knowledge, this is the first report of GPNMB^+^ Mph characterized by significant lipid metabolism and immunosuppressive activity in the IM. CellChat analysis revealed that GPNMB^+^ Mph in the IM exhibited prominent interactions with GZMB^+^ CD8^+^ T cells, predominantly mediated by HLA-CD8 engagement and the PTGES3–PTGER4 axis. Similar to the antigen-presenting TAMs in GBM that govern the TEX_prog to TEX_term transition [38], this specific ligation enables macrophages to reprogram CD8^+^ T cells toward immunosuppressive phenotypes.

Macrophages not only promote tumor cell growth but also induce epithelial–mesenchymal transition and secrete matrix metalloproteinases to facilitate tumor invasion and metastasis [42,43]. In glioblastomas, GPNMB^+^ Mph have been shown to alter tumor cell transcription programs, shifting them from a proneural to a mesenchymal state, thereby enhancing invasiveness and treatment resistance [32]. In this study, flow cytometry results from coculture models confirmed that GPNMB^+^ Mph suppressed the expression of cytotoxic molecules and induced CD8^+^ T cell exhaustion. Mechanistically, our multiomics and functional analyses revealed that GPNMB overexpression triggers lipid metabolic rewiring via the PI3K/AKT-COX2 cascade, culminating in elevated PGE2 secretion. This PGE2-mediated intercellular crosstalk provides a direct molecular explanation for the observed CD8^+^ T cell suppression. These results emphasize the key role of GPNMB^+^ Mph as an immune regulatory hub and provide a potential molecular target for novel drug development.

Nanoparticle technology has made significant progress in tumor therapy, particularly for solid tumors. Leveraging the intrinsic biological characteristics of tumors, nanoparticles can be engineered to specifically target tumor cells, enabling early cancer diagnosis, accurate localization, and ultimately precision therapy [44,45]. Recognizing the drawbacks of broadly depleting macrophages, namely, on-target off-tumor toxicity and loss of beneficial subsets, this study employed nanoparticle technology to achieve specificity within the TIME. Importantly, the APL_siGpnmb_ system minimizes off-target risks through a dual-layered restriction mechanism. At the cellular level, the CD206-targeted nanocarriers favor receptor-mediated endocytosis by M2-like TAMs. At the molecular level, the intrinsic sequence specificity of the siRNA payload acts as a secondary safeguard, rendering the payload biologically inert if internalized by non-GPNMB-expressing cells. Characterization of APL_siGpnmb_ confirmed it as a long-circulating, physically stable nanoplatform with active targeting potential. Furthermore, we validated that APL_siGpnmb_ specifically targeted and silenced GPNMB^+^ Mph in HCC tissues. In an in vivo mouse model, APL_siGpnmb_ effectively reversed the functional exhaustion of CD8^+^ T cells and inhibited tumor growth. Notably, the liver metastasis model that we established theoretically recapitulates the clinical features of patients with IM-HCC. The robust antitumor efficacy demonstrated by APL_siGpnmb_ highlights its significant translational potential, offering a promising strategy to improve HCC prognosis by specifically addressing immune cell reprogramming.​

In current clinical practice, advanced HCC is treated with tyrosine kinase inhibitors and immune checkpoint inhibitors. However, their efficacy is often limited by an immunosuppressive tumor microenvironment that drives treatment resistance [46]. Rather than replacing these standards of care, APL_siGpnmb_ is designed as a precision immunomodulator. By silencing GPNMB, it reverses local immunosuppression, promotes CD8^+^ T cell infiltration, and converts “cold” tumors into “hot” ones, thereby counteracting ICI resistance. This approach is well aligned with conversion therapy, offering a potential strategy for down-staging unresectable tumors or as a neoadjuvant/adjuvant intervention to reduce recurrence.

We acknowledge several limitations in this study. First, our internal scRNA-seq cohort was relatively small, including only 5 patients with IM-mHCC due to the clinical rarity of obtaining fresh matched tissues. While we rigorously validated the tissue preference of GPNMB^+^ macrophages using extensive, independent public scRNA-seq datasets across multiple metastatic cancer types, we clearly acknowledge that public data, which are well known for batch effects and differing tissue-processing protocols, cannot fully compensate for the lack of statistical power, clinical heterogeneity, and potential selection bias inherent to an internal cohort of just 5 people. The limited sample size also restricted our statistical power to evaluate sex-based or age-related demographic effects. Second, our current study focused exclusively on HBV-related, systemic therapy-naïve patients to establish a clear baseline for metastasis-driven microenvironmental changes. While this minimizes confounding variables, we recognize a limitation in generalizability, as almost no advanced HCC patients are treatment-naïve in clinical practice, and the incidence of NASH-related HCC is rapidly rising. Validating the conserved role of GPNMB^+^ macrophages in non-HBV-related HCC or postsystemic therapy settings remains a crucial priority. Third, due to technical challenges in acquiring sufficient viable tissue, we were unable to perform ex vivo functional assays using freshly isolated primary human cells; direct ex vivo validation remains a critical next step. Fourth, while murine models provide a valuable platform for intervention, species-specific differences in macrophage biology and immune organization represent inherent limitations, necessitating future studies in humanized models. Fifth, due to limited sample availability, we used CD39/TCF1 staining as surrogate markers to infer tumor reactivity and terminal exhaustion; direct TCR clonality assessment will be required to definitively confirm the clonal origin of GZMB^+^ CD8^+^ T cells. Furthermore, because the therapeutic nanoparticle targets CD206, which is broadly expressed on many different macrophage populations, the absence of direct blocking or cell-sorting experiments leaves a gap in our preclinical data regarding potential off-target toxicity on other macrophage subsets. Future studies utilizing specific fluorescent dyes to track nanoparticle delivery in vivo alongside cell-sorting assays will be necessary to definitively map the precise cellular uptake dynamics and rule out off-target effects. Finally, although our revision-based 1-month safety assessments demonstrated excellent systemic biocompatibility, ultralong-term longitudinal monitoring is required to definitively evaluate potential delayed toxicity or compensatory immune responses following APL_siGpnmb_ therapy.

In conclusion, this study reveals the distinct TIME characteristics of PL and IM within IM-mHCC. By revealing the immunosuppressive features of GPNMB^+^ Mph in the IM, our findings provide valuable insights for the development of more effective targeted therapies in IM-mHCC.

## Methods

### Patient tissue sample collection and follow-up

Ethical clearance for this research was obtained from the Zhujiang Hospital Ethics Committee (Southern Medical University). Patient eligibility was determined based on the following parameters: (a) meeting the histopathological criteria for IM-mHCC as defined by the Japanese Liver Cancer Study Group [17] and (b) having received no adjuvant therapy (including chemotherapy, radiotherapy, molecular targeted therapy, and immune checkpoint inhibitors) before surgery. Five patients with IM-mHCC were enrolled, and 15 tissue samples were collected, including matched PL, IM, and PCLT samples, which were subsequently subjected to scRNA-seq.

### Single-cell library construction and scRNA-seq

Detailed procedures for tissue dissociation, library preparation, and scRNA-seq are provided in the Supplementary Materials.

### Bioinformatic processing and scRNA-seq data interpretation

The computational workflow for scRNA-seq analysis involved initial quality control and preprocessing, followed by cell-type annotation and delineation of marker genes. To characterize cellular states, we performed pathway enrichment and calculated functional gene module scores. Furthermore, the study integrated single-cell regulatory network inference, clustering analysis, and developmental trajectory modeling. Transcription factor profiling, cell–cell communication analysis, and CNV estimation were also conducted to further dissect the tumor microenvironment. Detailed methodological parameters are provided in the Supplementary Materials.

### Multiplex IHC and multiplex immunofluorescence

The complete protocols are provided in the Supplementary Materials.

### Preparation and validation of APL_siGpnmb_

The experiments involving APL_siGpnmb_ nanoparticles included the preparation of APL_siGpnmb_, antibody thiolation, antibody conjugation, determination of conjugation efficiency, characterization of particle size and zeta potential, and morphological characterization. In vitro, the cytotoxicity of the nanoparticles was evaluated using Cell Counting Kit-8 assays, and their targeting specificity toward macrophages was validated by flow cytometry. In vivo, the liver-targeting specificity of systemically administered APL_siGpnmb_ was assessed in mouse HCC models using fluorescence live imaging. Further details on the experimental procedures are available in the Supplementary Materials.

### Cell function assays and animal experiments

Cell function assays included construction of macrophage models, quantitative real-time polymerase chain reaction, Western blot, flow cytometry, cell scratch assays, and transwell assays. Animal experiments included the establishment of HCC IM, orthotopic, and subcutaneous mouse models, as well as in vivo safety evaluation of the nanoparticles. The Supplementary Materials contain a comprehensive description of the experimental procedures.

Complete descriptions of other methods are documented in the Supplementary Materials.

### Statistical analysis

R (version 4.4.3) and GraphPad Prism (version 10.1.2) were used for statistical analyses. We performed Wilcoxon rank-sum test for 2-group comparisons and 1-way analysis of variance (ANOVA) for multiple-group comparisons. Kaplan–Meier analysis was used to generate survival curves, with comparisons made using the log-rank test. For single-cell transcriptomic data, Seurat was employed for core processing, while inferCNV was used to distinguish malignant cells. Advanced cellular dynamics and regulatory features were resolved through high-dimensional WGCNA for coexpression modules, CellChat for intercellular communication, and decoupleR for transcription factor activity. Cellular ontogeny and trajectories were reconstructed using Monocle2, scTour, and Slingshot. Additionally, NMF was utilized to identify tumor microenvironment metaprograms. Unless otherwise specified, statistical significance was defined as *P* < 0.05. Detailed algorithmic parameters are documented in the Supplementary Materials.

## Data Availability

The raw scRNA-seq and WES data generated in this study have been deposited in the Genome Sequence Archive (GSA-Human) at the National Genomics Data Center (NGDC), China National Center for Bioinformation / Beijing Institute of Genomics, Chinese Academy of Sciences, under accession codes HRA012230 and HRA017765, and are publicly accessible at https://ngdc.cncb.ac.cn/gsa-human. Processed scRNA-seq and WES data, integrative proteomic (DIA) and untargeted metabolomic profiles of fresh hepatocellular carcinoma tissues, as well as transcriptomic and metabolomic data derived from GPNMB-overexpressing THP-1 macrophages, have been deposited in the OMIX database (https://ngdc.cncb.ac.cn/omix) under accession numbers OMIX016781, OMIX010671, OMIX017090, and OMIX017088, respectively. Publicly available datasets used for cross-validation include TCGA-LIHC (https://portal.gdc.cancer.gov/), GSE238264, GSE149614, GSE169246, GSE306201, and GSE315534 (https://www.ncbi.nlm.nih.gov/geo/), alongside deep whole-genome sequencing data from the Chinese Liver Cancer Atlas (CLCA) accessed via cBioPortal (https://www.cbioportal.org/). All analysis code is available upon request.

## Supplementary Materials

Supplementary 1 Figs. S1 to S18 References [47–61]
