# Supplementary material for: Single-Cell Dissection of the Immune Microenvironment in Intrahepatic Metastasis of Multifocal Hepatocellular Carcinoma
Source: Research (Wash D C). 2026 Jul 27;9:1372. doi: 10.34133/research.1372 (PMC13402729; doi:10.34133/research.1372)
Supplement: Supplementary 1 — Figs. S1 to S18 References [47–61] [file research.1372.f1.docx]

**Single-Cell Dissection of the Immune Microenvironment in Intrahepatic Metastasis of Multifocal Hepatocellular Carcinoma**

Yuyan Xu^1^†, Cheng Zhang^1^†, Zhuocheng Ji^1^†, Chang Liu^4^†, Lei Cai^1^†, Chunming Wang^1^, Hangyu Liao^1^, Yaohong Wen^1^, Luhao Chi^1^, Chang Li^1^, Yucheng Huang^5^, Hongbo Guo^3*^, Qing Peng^2*^, Mingxin Pan^1*^

^1^ Second Department of Hepatobiliary Surgery, General Surgery Center, Guangdong Provincial Research Center for Artificial Organ and Tissue Engineering, Guangzhou Clinical Research and Transformation Center for Artificial Liver, Institute of Regenerative Medicine, Zhujiang Hospital, Southern Medical University, Guangzhou, China

^2^ Central Laboratory of The Second Affiliated Hospital, School of Medicine, The Chinese University of Hong Kong, Shenzhen & Longgang District People's Hospital of Shenzhen, Shenzhen, China

^3^ Neurosurgery Center, National Key Clinical Specialty, Engineering Research Center of Diagnostic and Therapeutic Technology and Devices for Cerebrovascular Diseases in Ministry of Education, Guangdong Provincial Key Laboratory on Brain Function Repair and Regeneration, Zhujiang Hospital Institute for Brain Science and Intelligence, Zhujiang Hospital, Southern Medical University, Guangzhou, China

^4^ School of Biomedical Sciences and Engineering, South China University of Technology, Guangzhou, China

^5^ PCFM Lab of Ministry of Education, School of Materials Science and Engineering, Sun Yat-sen University, Guangzhou, China

^†^ These authors contributed equally to the work

^*^Address correspondence to: Mingxin Pan; [panmx@smu.edu.cn](mailto:panmx@smu.edu.cn), Qing Peng; [pengqing@cuhk.edu.cn](mailto:pengqing@cuhk.edu.cn), and Hongbo Guo; [guohongbo911@126.com](mailto:guohongbo911@126.com).

**SUPPLEMENTARY MATERIALS**

**Supplementary methods**

**Tissue Sampling Strategies**

Matched tissues including the primary lesion (PL), intrahepatic metastasis (IM), and pericarcinomatous liver tissue (PCLT) were freshly obtained during surgical resection. To ensure the representativeness of the specimens and adequate capture of the tumor microenvironment, regional selection was meticulously guided by experienced hepatobiliary surgeons and pathologists. For PL and IM, tissue fragments (approximately 1 cm³) were selectively sampled from the macroscopically viable tumor periphery and invasive margins, deliberately avoiding necrotic cores, calcifications, and severely hemorrhagic areas. This strategy was designed to enrich for active tumor-immune cellular interactions. PCLT specimens were collected from normal-appearing liver parenchyma at least 2 cm away from the macroscopic tumor margin.

**Single cell library preparation**

All fresh tissue samples were immediately rinsed with phosphate-buffered saline (PBS) and stored at 4°C pending single-cell dissociation and subsequent single-cell RNA sequencing (scRNA-seq) library preparation. Single-cell sorting and library construction were performed following the MGI DNBelab C4 Single-Cell Transcriptome Experiment Protocol [47]. Following library construction, quantification was performed using a Qubit 4.0 Fluorometer, and library insert size distribution was assessed using Qsep 100 Bio-Fragment Analyzer. Libraries meeting quality specifications were subsequently subjected to cyclization and amplification to generate DNA nanoballs (DNBs). Qualified DNBs were sequenced on either DNBSEQ-T1 or DNBSEQ-T7 platforms.

**Whole exome sequencing**

Genomic DNA was extracted from different sample types using corresponding extraction kits, following the manufacturers' standard procedures. The purity of the extracted DNA was assessed using a NanoDrop spectrophotometer, and its concentration was measured by Qubit 3.0 Fluorometer. Agarose gel electrophoresis was performed to evaluate the integrity of DNA. Subsequently, 1 µg of DNA was subjected to library construction via enzymatic fragmentation. The library construction workflow encompassed DNA fragmentation and end repair, addition of a 3' adenosine overhang, adapter ligation, and PCR amplification to generate the pre-library. The concentration of the library was quantified using the Equalbit® dsDNA HS Assay Kit, and its fragment distribution was analyzed via Agilent 4200 TapeStation System. Eventually, the molar concentration of the library was accurately determined using the KAPA Library Quant kit (Illumina) universal qPCR Mix. Sequencing of the library was carried out on the NovaSeq X-Plus instrument with the corresponding kit.

**scRNA-seq data analysis**

In our study, we utilized the high-throughput single-cell RNA-seq technology of MGI’s DNBelab C4 platform. During the analysis, we employed the open-source software DNBelab_C_Series_scRNA-analysis-software provided by MGI to conduct quality control filtering, alignment, quantification, and cell identification of the raw data**,** ultimately obtaining a gene expression matrix for each cell. Subsequently, we imported the matrix into Seurat (v5.1.0) for downstream analysis. Low-quality cells were excluded based on the following criteria: (i) fewer than 300 detected genes; (ii) hemoglobin UMI counts > 5%; or (iii) mitochondrial gene content > 20% (thresholded to account for hepatocyte metabolic characteristics).

We performed log-normalization using the NormalizeData function and linear regression correction based on highly variable genes (HVGs) with the ScaleData function of Seurat [48]. To eliminate unexpected noise, we further filtered out mitochondrial genes, ribosomal genes, mitochondrial ribosomal genes, and heat shock protein genes. On the HVGs matrix, we carried out principal component analysis (PCA). Then, we integrated data from different samples using the IntegrateLayers function (method = ‘HarmonyIntegration’) [49] and visualized the data via UMAP or t-SNE.

Using the FindNeighbors function, we constructed a shared nearest neighbor graph and then used the FindClusters function (with a resolution of 0.8 for global clustering; for the second round of sub-clustering, the resolution was set to 1.3 for lymphoid cells and 0.6 for myeloid cells) to identify cell clusters. Differentially expressed genes (DEGs) for each cluster were calculated using the FindMarkers or FindAllMarkers function, p values were corrected using the Benjamini-Hochberg (BH) method. Based on canonical marker genes, we identified major cell types, including T cells, NK cells, B cells, myeloid cells, endothelial cells, fibroblasts, and epithelial cells. Then, we performed a second round of clustering separately on lymphoid and myeloid cells. The procedure for the second round of clustering was the same as the first.

**Tissue preference analysis**

To compare the distribution of cellular subpopulations across different tissues or groups, this study employed the methods outlined in reference [50, 51] to calculate the Odds Ratio (OR) and the ratio of observed over expected cell numbers Relative Over Expectation (Ro/e) for each cell cluster within diverse samples. Elevated Ro/e (Ro/e > 1) indicates a relative enrichment, while Ro/e < 1 indicates depletion. For odds ratio, values > 1.5 or < 0.5 were considered to indicate significant tissue preference or avoidance, respectively, consistent with previous studies.

**Single cell gene set enrichment analysis**

To identify significantly enriched biological pathways and functional gene sets within specific cell populations identified through clustering, we performed gene set enrichment analysis using the irGSEA R package (version 3.3.2) [52]. Briefly, irGSEA integrated several established single-cell GSEA methods. For this study, we primarily utilized the AUCell, UCell, singscore, ssGSEA, GSVA, AddModuleScore, JASMINE, and Viper method. We employed the MSigDB Hallmark, T cell state score [53] and macrophage function marker [27] from collection. Gene sets were considered significantly enriched if they passed a false discovery rate (FDR) adjusted p-value (padj) threshold of < 0.05. Functional landscapes were visualized using the built-in visualization functions of the irGSEA package.

**Trajectory inference and pseudotime analysis**

To decipher the dynamic processes of cell differentiation and state transitions, we employed three complementary algorithms for trajectory inference: Monocle 2, scTour, and Slingshot [54-56].

For Monocle 2 analysis, developmental trajectories of CD8^+^ T cell subsets were reconstructed using the Monocle 2 R package (v2.26.0). We utilized the differentialGeneTest function to identify genes that varied significantly across cell types (q-value < 0.01) as ordering genes. Dimensionality reduction was performed using the DDRTree algorithm (max_components = 2, num_dim = 6).

For the lineage analysis of monocyte/macrophage and CD8^+^ T cell subsets, the Slingshot R package (v2.2.0) was applied. The analysis was performed on the UMAP embedding specifically generated for myeloid cells. A cluster-based minimum spanning tree (MST) was constructed, and principal curves were fitted to identify developmental lineages with a shrink parameter of 0.2.

To further validate the directional transcriptomic flow of monocyte/macrophage differentiation using a deep learning framework, we employed the scTour Python package. The model was trained using the sct.train.Trainer with a negative binomial (nb) loss mode. Specific parameters for the variational autoencoder were configured as alpha_recon_lec = 0.5 and alpha_recon_lode = 0.5. After 400 epochs of training, we extracted the latent time and the latent space representation (X_TNODE). Vector fields (X_VF) were subsequently generated to visualize the directional differentiation flow from progenitor-like states toward mature macrophage sub-populations.

**Cell-cell communication analysis**

CellChat (v2.1.1) was utilized to analyze ligand-receptor interactions [57]. Briefly, the analysis began with the extraction and integration of the required cellular subpopulations for interaction analysis. An analysis object was created by integrating cell classification information using the createCellChat function. The analysis was performed using the CellChatDB.human database, with all parameters maintained at their default settings as specified by the software. For each group (e.g., PL and IM), over-expressed genes and L-R pairs were identified using the identifyOverExpressedGenes and identifyOverExpressedInteractions functions. The communication probability was computed using the computeCommunProb function with the triMean method. To facilitate comparative analysis, we merged the CellChat objects and utilized the compareInteractions and computeNetSimilarity functions to identify differential signaling pathways. Significant interactions were filtered based on a p-value threshold of < 0.05.

**Single cell transcription factor analysis**

To infer transcription factor (TF) activity from scRNA-seq data, we utilized the get_collectri function from the decoupleR package to obtain the human version of the CollecTRI network for analysis [58]. We then employed the Univariate Linear Model (ULM) method to predict the activity of each transcription factor within each cellular subpopulation.

**Single cell inference copy number variation**

To identify malignant cells, we used the inferCNV tool with default parameters to assess CNVs in epithelial cells. Epithelial cells from pericarcinomatous liver tissue (PCLT) were used as normal references.

**Single cell spatial transcriptomic analysis**

We performed fundamental analysis using Seurat's spatial transcriptomic pipeline. The SpaCET package's SpaCET [59]. GeneSetScore function calculated feature scores for GPNMB_TAMs and GZMB_CD8T cells in each spot. Gene set scores were visualized with SpaCET.visualize.spatialFeature.

**Cellular program identification via non-negative matrix factorization**

To delineate recurrent cellular programs within the tumor microenvironment (TME), we initially computed the fractional abundance of each major TME cellular category (excluding epithelial cells) per tumor specimen. This generated composition matrix V (features: cell types, observations: samples). Subsequent application of non-negative matrix factorization (NMF) enabled systematic identification of co-varying cellular programs [60].

**hdWGCNA analysis of malignant cells**

To infer, analyze, and interpret gene co-expression networks in malignant cells, we performed high-dimensional WGCNA (hdWGCNA) on transcriptomic data [61]. A soft power threshold of 9 was selected for network construction, with all other parameters retained at default settings for subsequent analysis.

**Multiplex immunohistochemical (mIHC) staining**

Multiplex immunohistochemical (mIHC) staining was performed using nine primary antibodies applied sequentially. Tissue sections were subjected to heat-induced epitope retrieval (HIER) under high pressure (120°C) for 5 minutes. Endogenous peroxidase activity was quenched using peroxidase blocking solution (Beyotime, Cat. No. P0100A) for 15 minutes at room temperature, followed by protein blocking with immunohistochemical blocking buffer (Biosharp, Cat. No. BL736A) for 1 hour. Primary antibodies were applied sequentially at a dilution of 1:200–1:500: GPNMB (Abcam, Cat. No. EPR20004-39), CD68 (ZSGB-BIO, Cat. No. KP1), CD8 (ZSGB-BIO, Cat. No. SP16), PDCD1 (ZSGB-BIO, Cat. No. UMAB199), GZMB (ZSGB-BIO, Cat. No. EPR22084-58), MKI67 (Proteintech, Cat. No. SP6), CD163 (Proteintech, Cat. No. 16646-1-AP), DOCK4 (Proteintech, Cat. No. EPR14302), and C1QA (Proteintech, Cat. No. EPR19929). Sections were incubated at 4°C overnight (or at room temperature for 60 minutes), followed by HRP-conjugated secondary antibody incubation and signal detection using AEC chromogenic substrate (Solarbio, Cat. No. A2010-10) for 5–10 minutes. Between each step, sections were washed three times with TBST (0.1% Tween-20) for 5 minutes. Whole-slide imaging was performed using the Pannoramic MIDI II system (3D Histech, Hungary). For subsequent staining rounds, the AEC precipitate was removed by washing with 95% ethanol, followed by antibody stripping using antibody stripping solution (Beyotime, Cat. No. P1453) and a second round of HIER (5 minutes) to prepare for the next antibody incubation cycle.

**Multiplex immunofluorescence (mIF) staining**

Multiplex immunofluorescence (mIF) staining was conducted using the Opal 7-Color TSA kit (Akoya Biosciences, Cat. No. NEL797001KT) following a sequential staining protocol. Sections were first subjected to HIER for 5 minutes, then blocked with Antibody Diluent/Block for 10 minutes. Primary antibodies were applied in four sequential rounds: (1) GPNMB (Abcam, Cat. No. EPR20004-39); (2) CD68 (ZSGB-BIO, Cat. No. KP1); (3) a mixture of GZMB (ZSGB-BIO, Cat. No. EPR22084-58) and PDCD1 (ZSGB-BIO, Cat. No. UMAB199) (only if raised in different host species; otherwise applied sequentially); and (4) CD8 (ZSGB-BIO, Cat. No. SP16). Each round consisted of primary antibody incubation (1 hour at room temperature), HRP-conjugated secondary antibody incubation (10 minutes), tyramide signal amplification using Opal fluorophores (Akoya), and antibody stripping via HIER (5 minutes) to remove the immune complex before the next round. After completion of all four rounds, sections were counterstained with DAPI (Akoya Biosciences, Cat. No. FP1490) for 5 minutes at room temperature. Multispectral whole-slide imaging was acquired using the Pannoramic MIDI II multispectral imaging system (3D Histech, Hungary).

**Preparation of APL_siGpnmb_ nanoparticle**

siGpnmb aqueous solution (40 μg in 10 μL) was added to a DMF solution containing DOTAP (0.2 mg) and DLin-MC3-DMA (0.2 mg), followed by mixing under continuous vortexing. The mixture was allowed to stand at room temperature for 10 minutes. Subsequently, 250 μL of DMF containing Mal-PEG-PLGA (5 mg) was added. After thorough mixing, the solution was added dropwise into 5 mL of DEPC-treated water under constant magnetic stirring. Following 1 hour of agitation, the nanoparticles were transferred to a 15 mL ultrafiltration tube (MWCO 100 kDa) and washed three times with DEPC water to remove the organic solvent DMF. The nanoparticle suspension was concentrated to 1 mL and stored at 4°C.

**Antibody thiolation and conjugation**

CD206 antibody (Proteintech, Cat. No. 18704-1-AP) was incubated with Traut's reagent(2-iminothiolane) at a 1:20 molar ratio at room temperature in the dark for 30 minutes (not exceeding 1 hour). The mixture was then transferred to an ultrafiltration centrifuge tube (MWCO 50 kDa) and centrifuged at 3,600 rpm for 10 minutes; this washing step was repeated three times to obtain 300 μL of thiolated CD206 antibody solution. The resulting 300 μL of thiolated antibody was added to 1 mL of nanoparticle suspension and incubated on an orbital shaker at room temperature in the dark for 18 hours. The mixture was subsequently transferred to an ultrafiltration tube (MWCO 300 kDa), washed with PBS, and centrifuged at 4,000 rpm for 5 minutes (repeated three times). The final nanoparticle suspension was concentrated to 1 mL and stored at 4°C.

**Determination of antibody coupling rate**

The BCA protein concentration assay kit was used to measure the CD206 content on the surface of APL_siGpnmb_. 3 mL of BCA reagent A and 60 μL of BCA reagent B were mixed to form 3 mL of BCA working solution. Standard protein solutions at concentrations of 0, 0.025, 0.05, 0.1, 0.2, 0.3, 0.4 and 0.5 mg/mL were prepared and mixed with 200 μL of BCA working solution. After incubation at 37℃ for 20 minutes, the absorbance at 562 nm was measured using a microplate reader to plot a standard curve. For APL_siGpnmb_, 10 μL was diluted with 10 μL of PBS and then mixed with 200 μL of BCA working solution. The absorbance was measured as per the kit's instructions, and the CD206 antibody concentration was determined using the standard curve. PL_siGpnmb_ was used as a control to calculate the coupling rate of CD206 antibodies on APL_siGpnmb_. The coupling rate was calculated using the formula: (APL_siGpnmb_ antibody amount - PL_siGpnmb_ antibody amount)/input antibody amount.

**Determination of particle size and potential**

APL_siGpnmb_ nanoparticles were diluted to 1 mg/mL. An appropriate volume was added to the instrument's cuvette, and the average hydrodynamic particle size, polydispersity coefficient and potential were measured using a Malvern dynamic light scattering instrument with water as the dispersing medium. The measurement parameters were: 35 mW He-Ne laser (633 nm), scattering angle of 90°, and temperature of 25℃.

**Morphological characterization**

APL_siGpnmb_ solution was diluted to 0.5 mg/mL. 20 μL was placed on a copper grid using a syringe and air-dried in a laminar flow hood. The morphology of the particles was observed using transmission electron microscopy.

**CCK8 assay for cytotoxicity detection**

Following preparation of single-cell suspensions, RAW264.7 cells treated with various concentrations of APLsiGPNMB nanoparticles were dispensed into 96-well plates at a density of 1,000 cells per well in 100 μL of culture medium supplemented with 5% serum using a multichannel pipetting system. Five replicate wells were established for each treatment condition daily throughout the 7-day culture period. At designated time points each day, the culture medium was aspirated and replaced with CCK-8 working solution (Beyotime, Cat. No. C0038,prepared by diluting CCK-8 stock solution 1:10 in 5% serum-containing culture medium). Following 2 hours of incubation, absorbance was measured using a multimode microplate reader, and proliferation kinetics were assessed through continuous time-course monitoring.

**Construction of macrophage model**

Cationic lipid transfection reagent was mixed with GPNMB recombinant plasmid DNA in serum-free medium and incubated at room temperature for 15 minutes to form neutral complexes. The complex solution was then slowly added to the 293T cell culture system and incubated in a constant-temperature incubator. Culture supernatants containing viral particles were collected 48 and 72 hours post-transfection. The viral suspension was added to THP-1 and RAW264.7 cells in the presence of suspension cell-specific transduction enhancer (Genechem, Cat. No. REVG006) at a 1X working concentration. After 8 hours of incubation, the culture medium was replaced. Transduction efficiency was subsequently evaluated via inverted fluorescence microscopy, and successfully transduced cells were continuously selected using puromycin (LEGENE, Cat. No. CA0069), the antibiotic corresponding to the resistance gene.

**Validation of GPNMB expression by qRT-PCR**

Total RNA was extracted from THP-1/RAW264.7 cells using the Trizol method（Vazyme，Cat. No. R401-01）, and qRT-PCR was performed to detect GPNMB expression. The relative expression level of GPNMB was analyzed using the 2^−ΔΔCt^ method.

**Validation of GPNMB expression by western blotting**

Proteins were extracted from lentivirus-infected THP-1 and RAW264.7 cells, subjected to SDS-PAGE, and transferred onto membranes via wet transfer at a constant current of 200 mA. The membranes were incubated overnight at 4°C with primary antibodies against β-actin (Proteintech, Cat. No. 66009-1-Ig) and GPNMB (Abmart, Cat. No. M069478), followed by incubation with corresponding secondary antibodies (Proteintech, SA00001-1 and SA00001-2). Finally, the signals were detected using enhanced chemiluminescence reagent (Thermo Fisher, Cat. No. 34577) on a Tanon chemiluminescence imaging system.

**Cell scratch assay**

When cells fully covered the bottom of the well, a straight scratch was made using a 200-μL sterile pipette tip. Cells were rinsed three times with PBS to remove detached cells. Wound healing was observed and photographed at 0, 12, and 24 hours post-scratch using an inverted microscope at the same magnification and focal length. ImageJ was used to measure wound widths, and cell migration rate was calculated as: Cell migration rate (%) = {1 - [wound width (12 h or 24 h)/wound width (0 h)]} × 100%. Each experiment was repeated at least three times.

**Transwell assay**

Cells were resuspended in phenol-red free DMEM and added to the upper chamber of the Transwell insert, with chemoattractant medium in the lower chamber. After 24 hours of incubation, the migrated cells were fixed using cell fixation solution (Beyotime, Cat. No. P0099) and subsequently stained with crystal violet. The surface was then gently rinsed with running water to remove residual particles and wiped clean.

**Cell scratch assay**

THP-1 cells were seeded at a density of 5 × 10⁵ cells per well in a 6-well plate and treated with PMA (Merck, Cat. No. P1585) in complete medium (RPMI 1640 supplemented with 10% FBS) for 48h to induce macrophage differentiation. After adhesion, the medium was aspirated, and adherent cells were gently washed with phosphate-buffered saline (PBS) to remove residual PMA and non-adherent cells. The resulting macrophage-derived THP-1 cells were then cultured in fresh complete medium containing IL-4 (Thermo Fisher Scientific, Cat. No. 200-04) and IL-13 (Thermo Fisher Scientific, Cat. No. 200-13) for an additional 48 h to polarize into M2 macrophages.

For RAW264.7 cells (a murine macrophage cell line), cells were seeded at appropriate density (approximately 2 × 10⁵ cells per well in a 6-well plate) and allowed to adhere overnight. M2 polarization was induced by treating adherent cells with IL-4 (Thermo Fisher Scientific, Cat. No. 214-14) or IL-10 (Thermo Fisher Scientific, Cat. No. 210-10) in complete medium for 48 h, without PMA pretreatment.

**Flow cytometry**

Single-cell suspensions were prepared from co-culture models and stained separately for human (THP-1/PBMC) and murine(RAW264.7)systems using the following antibody panels: Human THP-1/PBMC co-culture system: Cells were first incubated with Fixable Viability Stain FVS620 (BD Horizon, Cat.No.564407) for 15 minutes at room temperature in the dark to exclude dead cells. Surface staining was performed by incubating cells with the following antibody cocktail for 30 minutes at room temperature protected from light: CD3-PerCP-Cy5.5 (BD, Cat.No.332771), CD45-AF700(BD, Cat.No.557943), CD8-APC-Cy7 (BD, Cat.No.557834), PD-1-BV510 (BD, Cat.No.563093) , and TIM-3-PE-Cy7 (BD, Cat.No.565566). Cells were then washed twice with staining buffer (PBS + 2% FBS) to remove unbound antibodies.

For intracellular staining of cytotoxic markers, cells were fixed and permeabilized using Cytofix Fixation solution (BD Biosciences, Cat.No.554714) for 1 hour at 4°C. After fixation, cells were washed twice with Wash Buffer (provided in Cat.No.554714 kit) and incubated with intracellular antibody cocktail for 30 minutes at room temperature protected from light: Granzyme B-BV421 (BD,Cat.No.563462), IFN-γ-BV650 (BD,Cat.No.563726), and Perforin-AF647 (BD,Cat.No.563689). Cells were subsequently washed twice with Perm/Wash Buffer and resuspended in staining buffer for flow cytometric analysis on a BD LSRFortessa™X-20.

Murine RAW264.7 co-culture system: Cells were first stained with Fixable Viability Stain FVS620(BD Horizon, Cat.No.564407) for 15 minutes. Surface staining was performed with: CD3-BV510 (BD, Cat.No.563024), CD45-AF700 (BD, Cat.No.557939), CD8-APC-Cy7 (BD, Cat.No.557654), PD-1-BV605 (BD, Cat.No.563111), and TIM-3-BV711 (BD, Cat.No.565763) for 30 minutes at room temperature in the dark, followed by washing with staining buffer.

For intracellular staining, cells were processed using Cytofix/Cytoperm Kit (BD Biosciences, Cat.No.554714) as described above. Cells were then incubated with: IFN-γ-PE (BD, Cat.No.554412), Granzyme B PE-Cy7 (BD, Cat.No.560808) , and Perforin-APC (Thermo Fisher, Cat.No.17-9392-42) for 30 minutes at room temperature protected from light. After washing with Wash Buffer, cells were resuspended for analysis.

**Splenic injection model of liver metastasis**

Ten 4-to5-week-old SPF-grade male C57BL/6 mice were subjected to intrasplenic injection of HEP534 murine hepatocellular carcinoma cells (1×10⁶ cells per mouse). The detailed surgical procedure was performed as follows: Following skin preparation, disinfection, and laparotomy, the splenic pedicle was temporarily occluded using 4-0 surgical silk sutures. A 30-gauge needle was inserted via a subcapsular route along the dorsal surface of the spleen near its inferior pole, and 100 μL of tumor cell suspension was slowly injected over 30–60 seconds. Successful intrasplenic delivery was confirmed by immediate visualization of localized pallor, swelling, and vascular blanching at the injection site. The needle was withdrawn after restoration of normal splenic coloration. Hemostasis was achieved by applying gentle pressure with a medical cotton swab soaked in normal saline to the injection site for 2 minutes. Five minutes thereafter, splenectomy was performed starting from the superior pole using electrocautery, with maximal excision of the main vascular trunk and adjacent adipose tissue. Following repositioning of the abdominal viscera, the surgical incision was closed in layers and disinfected. Intrasplenic tumor engraftment was subsequently confirmed by small-animal in vivo imaging on postoperative days 5–7.The mice were then randomly divided into two groups (n=5/group): Control group (PBS) and Experimental group (APL_siGpnmb_, 1 mg/kg). Treatments were administered via tail vein injection (100 μL) every two days for a total of five injections. The experiment was terminated 24 hours after the last treatment. Liver and lung tissues were collected, and hepatic metastasis was assessed by H&E staining. The experiment was prematurely terminated if severe cachexia occurred. All procedures were performed in compliance with animal ethics guidelines. Isoflurane inhalation anesthesia was used, and postoperative analgesic care was provided.

**Subcutaneous HCC mouse model**

Twenty 4-to5-week-old SPF-grade male C57BL/6 mice were inoculated subcutaneously with Hep1-6 cells. Tumor size was measured every other day. When the tumor volume reached 80 ± 20 mm³, mice were randomized into groups (n = 5/group). The treatment groups were as follows: Control group (PBS), Experimental group 1 (APL_siGpnmb_, 1 mg/kg), Experimental group 2 (PD-1, 10 mg/kg), and Combination group (APL_siGpnmb_ + PD-1). Treatments were administered via tail vein injection (100 μL) every two days for a total of five injections. On day 14 or when the tumor volume exceeded 1500 mm³, tumors were excised and measured.

**Orthotopic HCC mouse model**

Twenty C57BL/6 mice were inoculated with Hep1-6 cells stably expressing luciferase in the left lateral liver lobe. Tumor size was monitored every other day using in vivo bioluminescence imaging. When the tumor signal reached 1×10⁶ photons/sec, mice were randomized into treatment groups (n = 5/group) as in the subcutaneous model. Bioluminescence imaging was performed every 48 hours. After five treatments, tumors were excised and weighed.

**In vivo safety evaluation of APL_siGpnmp_**

The safety of nanoparticles was assessed by dynamically monitoring body weight, evaluating liver and kidney function, assessing hematopoietic system function, and examining pathological changes in mouse organs. Blood was collected via retro-orbital sinus puncture for liver and kidney function tests (AST, ALB, TBIL, ALP, UREA, CREA, UA) and hematopoietic function tests (RBC, HGB, WBC and its subpopulations, PLT). Pathological changes in the spleen and kidneys were assessed using H&E staining, and whole-field images were captured with a panoramic digital slide scanning system.

**Animal in vivo imaging**

Starting from day 7 post-inoculation, C57BL/6 mice implanted with luciferase-expressing tumor cells were subjected to longitudinal monitoring via in vivo bioluminescence imaging using the Bruker In-Vivo F PRO small-animal imaging system. Prior to image acquisition, mice were administered intraperitoneally with D-luciferin potassium salt (VAZYME, Cat. No. DD1210) working solution at a dosage of 150 mg/kg (diluted to 15 mg/mL in sterile PBS) 15 minutes before imaging. Animals were subsequently placed in an anesthesia induction chamber and maintained under 2% isoflurane anesthesia, then transferred to the imaging platform and secured in the prone position. The field of view was adjusted to appropriate parameters, with exposure time set to either Auto or a fixed duration of 30–60 seconds, and detection performed using an Open Filter configuration to acquire bioluminescence signal intensity from the tumor region.

**Supplementary Figures**

**
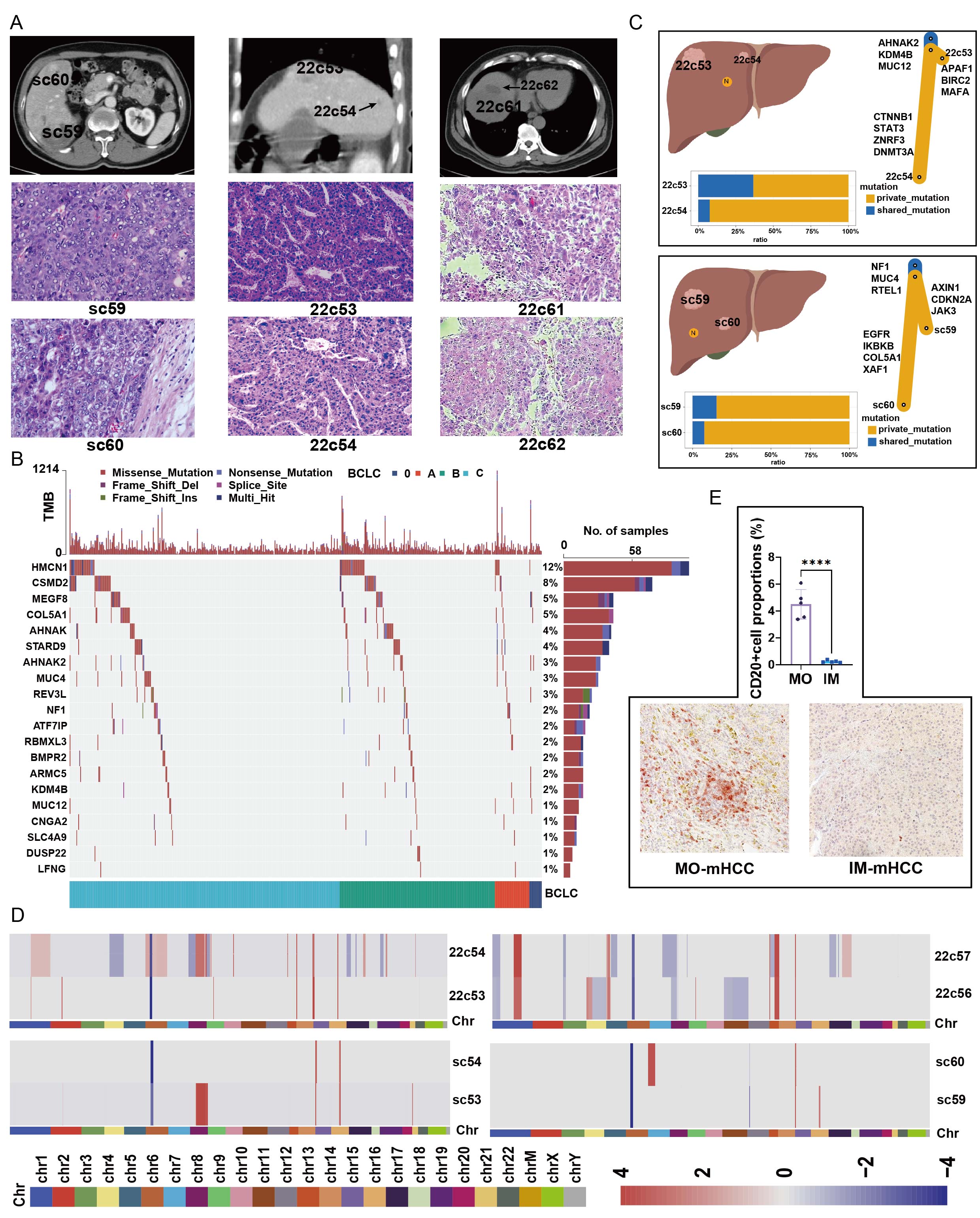
**

**Supplementary Figure 1. Genomic landscape and single-cell transcriptome profiling of IM-mHCC.**

(A) Representative cases of IM-mHCC showing CT imaging and histopathological features.

(B) Mutational frequency landscape of the key genes identified in our cohort, mapped across patients from the Chinese Liver Cancer Atlas (CLCA) dataset.

(C) Spatial distribution of samples showing the percentage of shared and private somatic mutations across tumor regions from each patient. Corresponding phylogenetic trees are also displayed.

(D) Copy number variation (CNV) landscape in IM-mHCC patients (red: gain; blue: loss).

(E) CD20 immunohistochemical staining in IM-mHCC versus MO-mHCC patients.

Abbreviations: IM-mHCC, intrahepatic metastatic multifocal hepatocellular carcinoma; MO-mHCC, multicentric occurrence multifocal hepatocellular carcinoma; CLCA, Chinese Liver Cancer Atlas; CNV, copy number variation; CT, computed tomography.


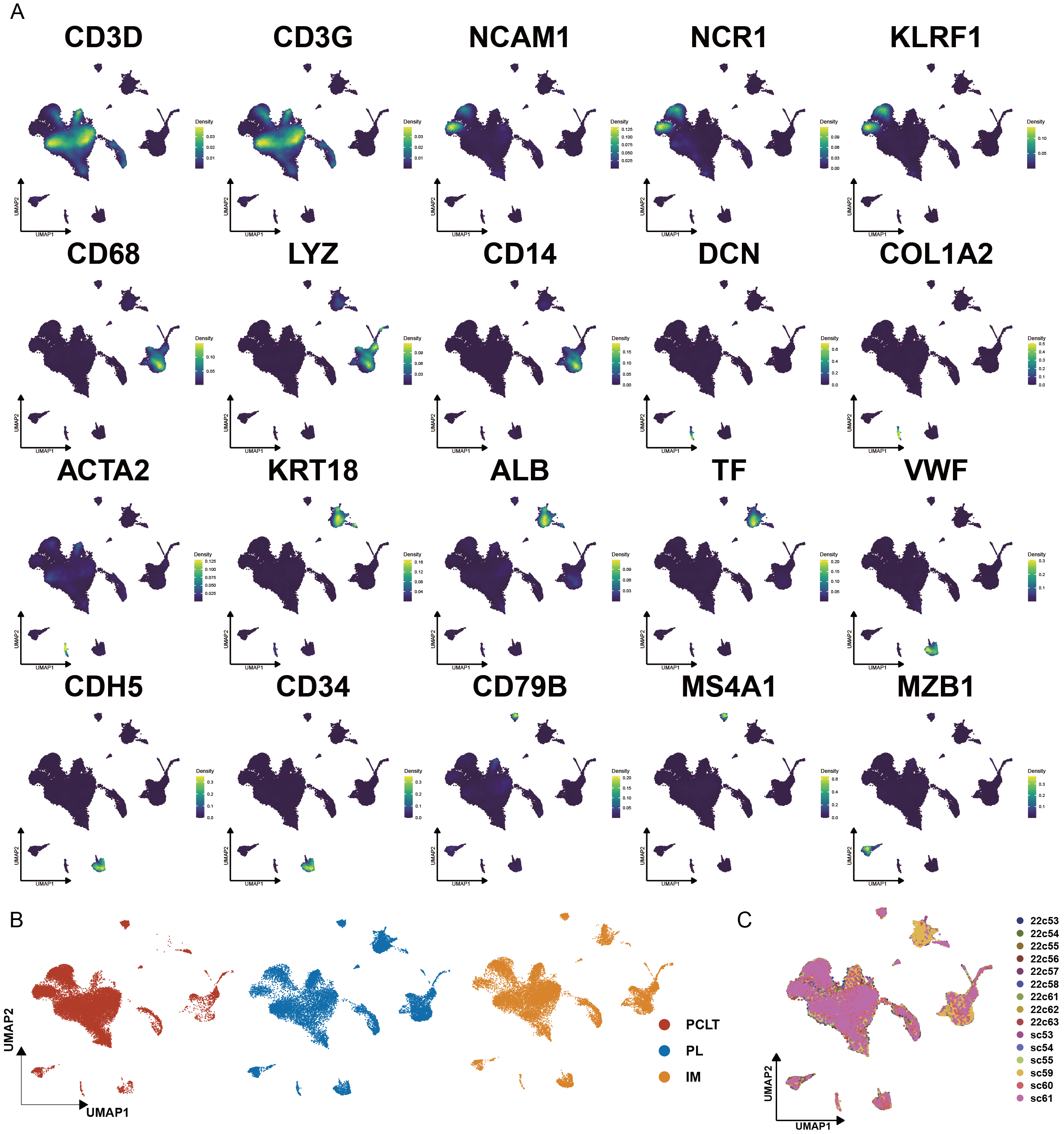


**Supplementary Figure 2. Cellular origin and identity visualization in IM-mHCC.**

(A) UMAP density plots illustrating the expression of canonical marker genes used to identify major cell lineages.

(B) UMAP visualization of all cells, splited by tissue origin: pericarcinomatous liver tissue (PCLT, red), primary lesion (PL, blue), and intrahepatic metastasis (IM, orange).

(C) UMAP visualization of cells by sample origin.

Abbreviations: UMAP: Uniform Manifold Approximation and Projection; PCLT: pericarcinomatous liver tissue; PL: primary lesion; IM: intrahepatic metastasis; mHCC, multifocal hepatocellular carcinoma.


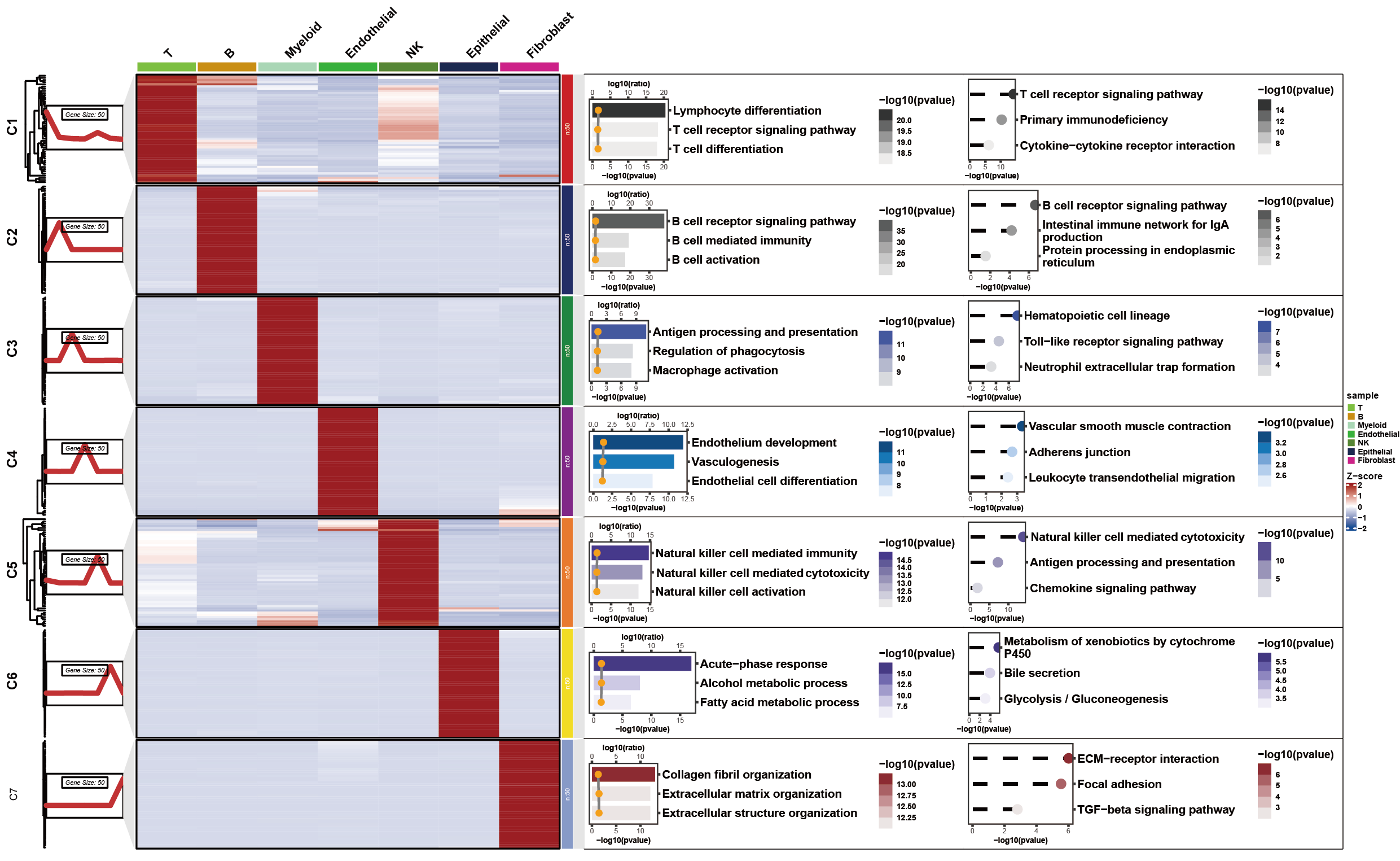


**Supplementary Figure 3. Functional landscape of major cell lineages in IM-mHCC.** (Left) Heatmap displaying the expression profiles of the top 50 upregulated genes across the seven major cell types. The color scale represents the row-normalized Z-score of gene expression levels.

(Middle) Bar plots showing significantly enriched Gene Ontology (GO) biological processes for each cell cluster.

(Right) Dot plots visualizing representative KEGG signaling pathways enriched in each cell type.

Abbreviations: GO, Gene Ontology; KEGG, Kyoto Encyclopedia of Genes and Genomes.

**
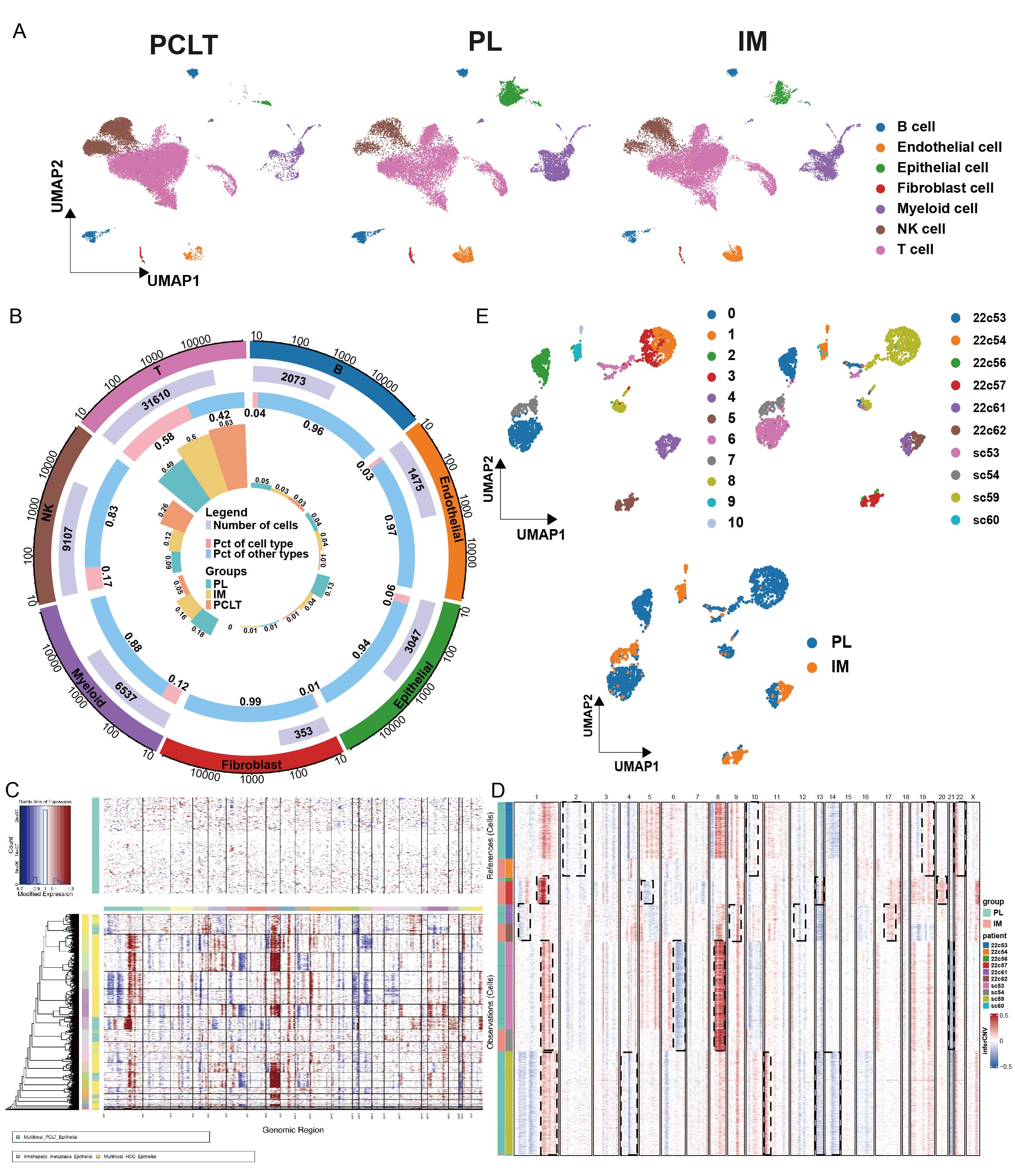
**

**Supplementary Figure 4. Single-cell Profiling of PL and IM reveals cellular heterogeneity, CNV landscape, and clonal evolution.**

(A) UMAP plots stratified by tissue origin, with colors denoting distinct cell types.

(B) Multi-circle plot showing cell counts of major subtypes (outer), their proportions among all cells (middle), and tissue-type-specific cellular composition (inner).

(C) Single-cell CNV landscape of epithelial cells inferred by the inferCNV algorithm. Malignant cells were identified based on large-scale chromosomal alterations compared to the diploid reference cells from PCLTs.

(D) CNV landscape demonstrating concordant evolution between primary and metastatic lesions.

(E) UMAP visualization of the sub-clustered malignant cells (top-left), colored by sample identity (top-right) and tissue origin (bottom).

Abbreviations: CNV, copy number variation; PCLT, pericarcinomatous liver tissue; IM, intrahepatic metastasis; PL, primary lesion; UMAP, uniform manifold approximation and projection.


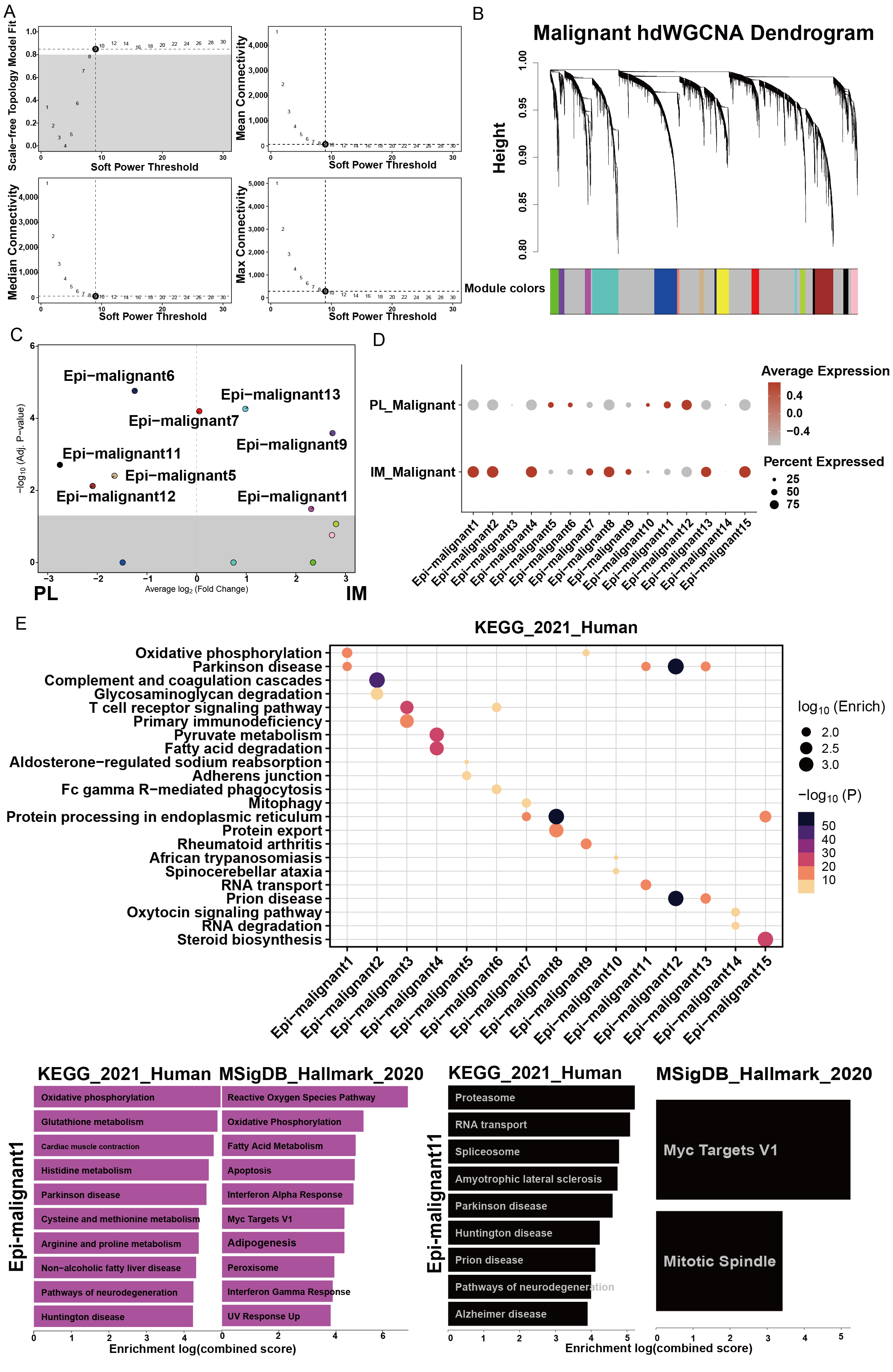


**Supplementary Figure 5. High-dimensional WGCNA (hdWGCNA) identifying co-expression modules and functional landscapes of malignant cells.**

(A) Optimization and selection of the soft-thresholding power (β) for network construction.

(B) The hdWGCNA dendrogram displaying the co-expression modules. Each leaf represents a gene, and the colors indicate the module assignment.

(C) Volcano plot visualizing differential module eigengenes (DMEs) in IM vs. PL.

(D) Dot plot showing the enrichment of co-expression modules in malignant cells from primary and metastatic lesions.

(E) Bubble plots visualizing the top two enriched terms per module from the KEGG 2021 Human (top) databases. Bubble size indicates the enrichment level, and color denotes significance. Bar plots demonstrating the functional enrichment of core genes in modules 1 (bottom left) and 11 (bottom right) for the KEGG and MSigDB Hallmark databases.

Abbreviations: hdWGCNA, high-dimensional weighted gene co-expression network analysis; DME, differential module eigengene; PL, primary lesion; IM, intrahepatic metastasis; KEGG, Kyoto Encyclopedia of Genes and Genomes; MSigDB, Molecular Signatures Database.


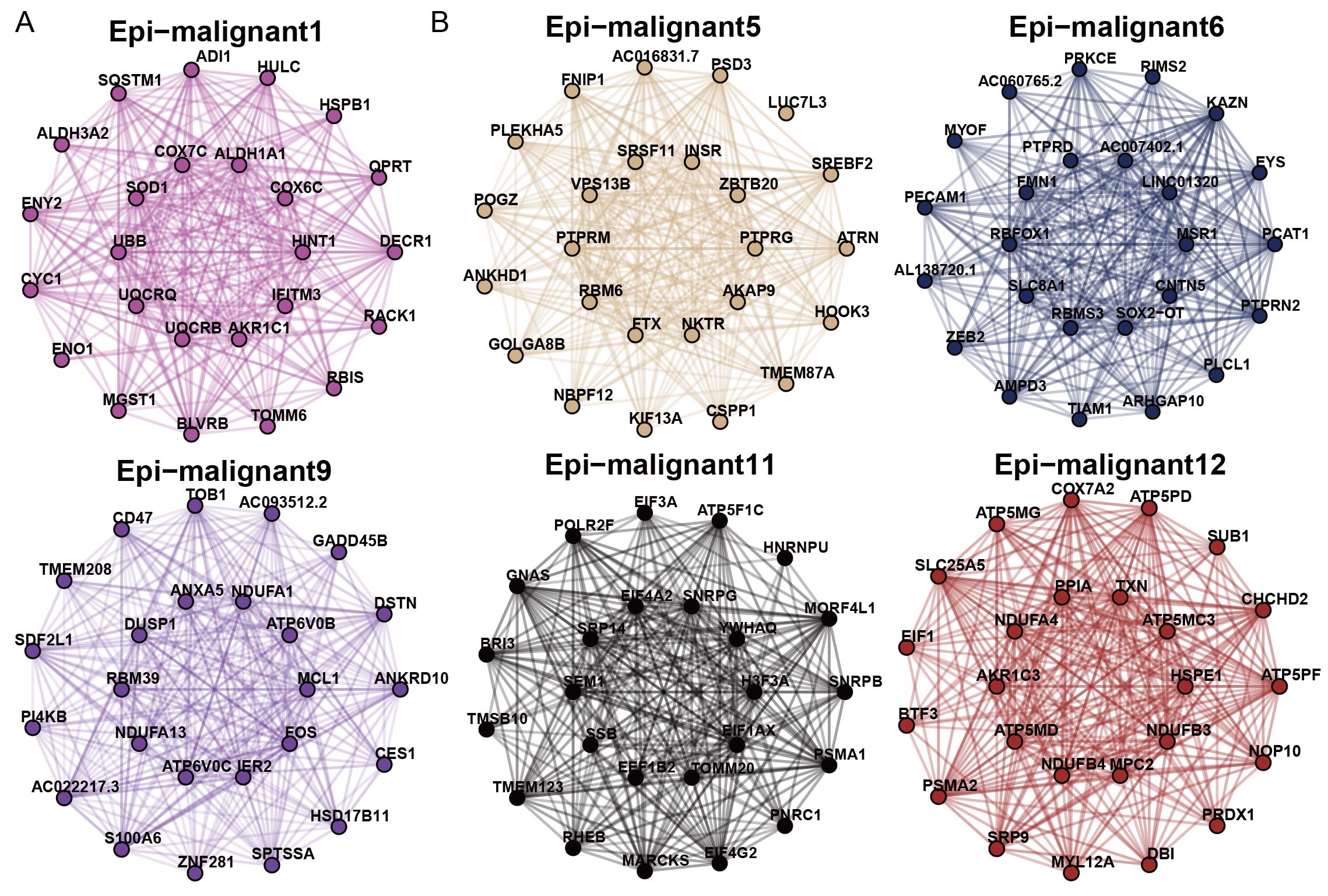


**Supplementary Figure 6. Intra-modular hub gene networks for IM- and PL-enriched malignant modules.**

(A) Top 25 hub gene network of upregulated modules in IM (e.g., Epi-malignant1, Epi-malignant9). For each network, the top 10 core hub genes (based on intra-modular connectivity) are positioned centrally, while the remaining 15 peripheral genes are arranged in the outer ring.

(B) Top 25 hub gene network of upregulated modules in PL (e.g., Epi-malignant5, Epi-malignant6, Epi-malignant11, Epi-malignant12).

Abbreviations: IM, intrahepatic metastasis; PL, primary lesion.


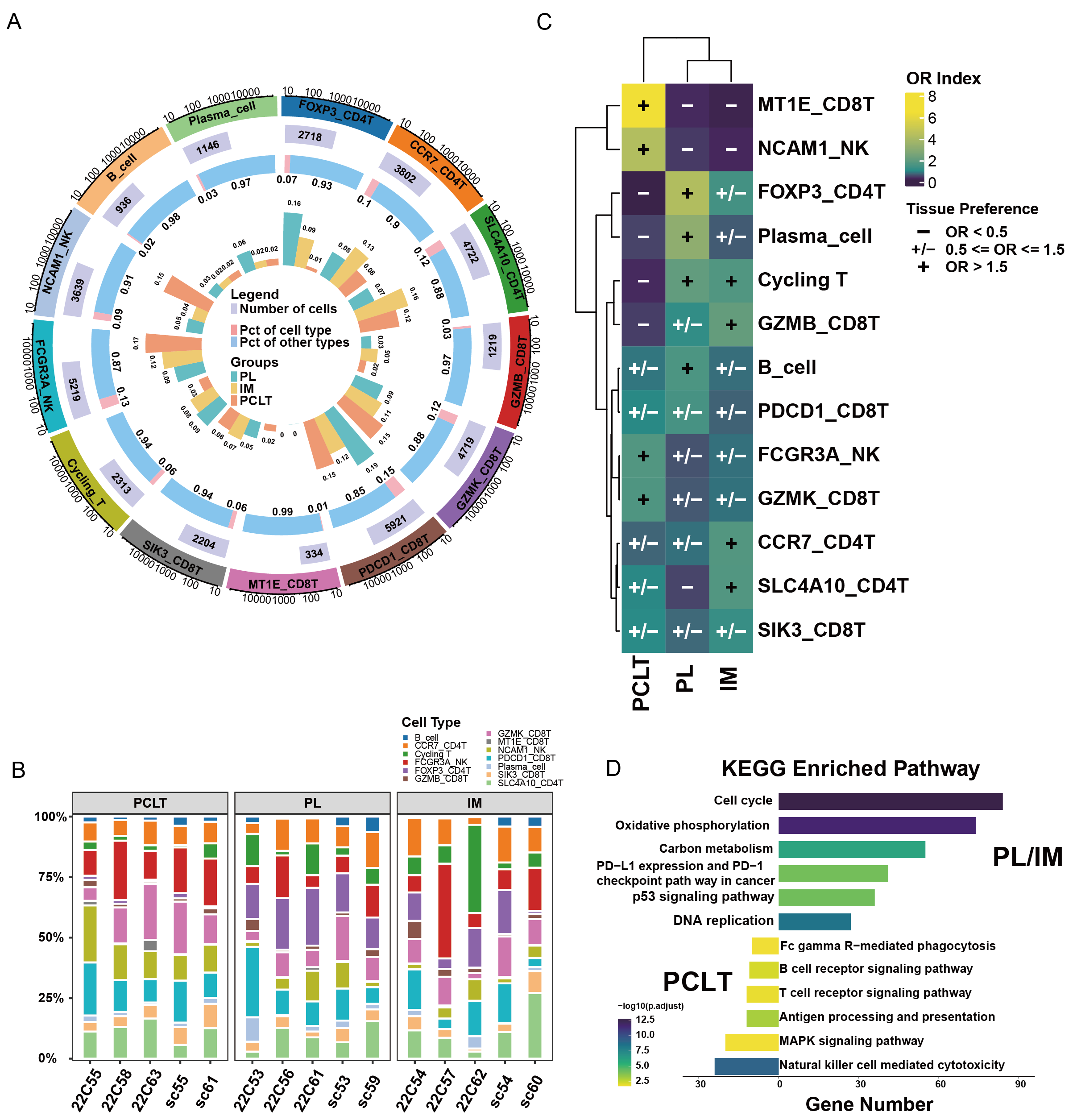


**Supplementary Figure 7. Comprehensive transcriptomic and functional landscape of lymphoid cell subsets.**

(A) Multi-circle plot showing cell counts of lymphoid subsets (outer), their proportions among all cells (middle), and tissue-type-specific cellular composition (inner).

(B) Stacked bar plot showing the proportional distribution of lymphoid cell subsets across individual patient samples, stratified by tissue origin (PCLT, PL, and IM).

(C) Heatmap of OR indices for lymphoid subsets across the three tissue origins. Odds ratio (OR) > 1.5 indicates enrichment and OR < 0.5 indicates depletion.

(D) KEGG pathway enrichment analysis comparing T cell functions between PL/IM and PCLT.

Abbreviations: KEGG, Kyoto Encyclopedia of Genes and Genomes; PCLT, pericarcinomatous liver tissue; PL, primary lesion; IM, intrahepatic metastasis; OR, odds ratio.


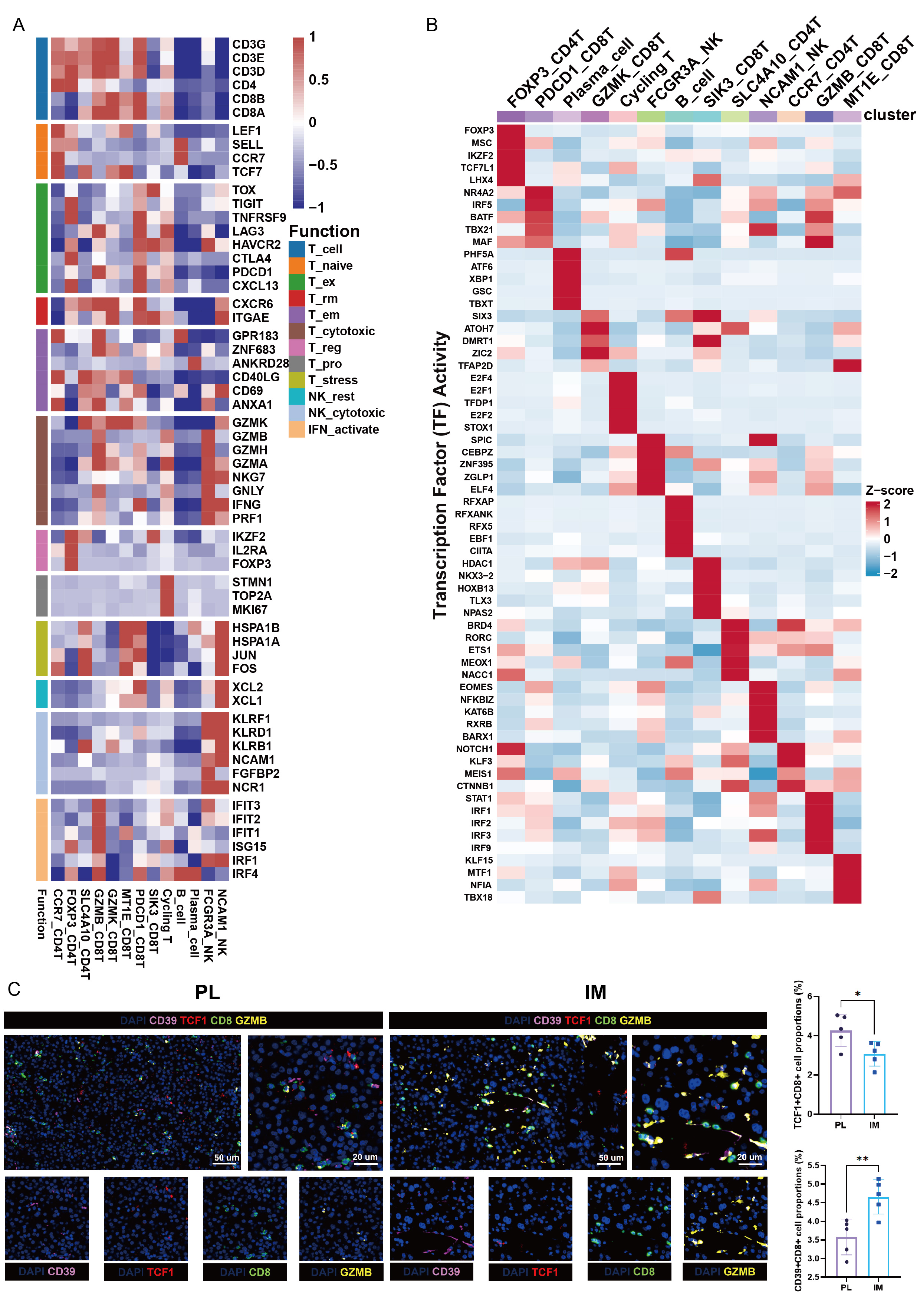


**Supplementary Figure 8. Transcriptional profiles, transcription factor activity, and exhaustion status validation of lymphoid subsets.**

(A) Heatmap depicting the normalized expression of canonical NK/T-cell marker genes across lymphoid cell subsets.

(B) Heatmap displaying the regulon activity scores for top-ranking transcription factors across lymphoid subsets.

(C) Representative mIF images illustrating the expression and spatial distribution of CD39, TCF1, CD8, and GZMB in the PL and IM. Bar graphs on the right display the quantification of TCF1^+^ and CD39^+^CD8^+^ cell proportions in the respective regions. Scale bars: 50 μm and 20 μm. Data are presented as mean ± SEM. *P < 0.05, **P < 0.01.

Abbreviations: mIF, multiplex immunofluorescence; PCLT, pericarcinomatous liver tissue; PL, primary lesion; IM, intrahepatic metastasis; Ro/e, ratio of observed to expected cell numbers.


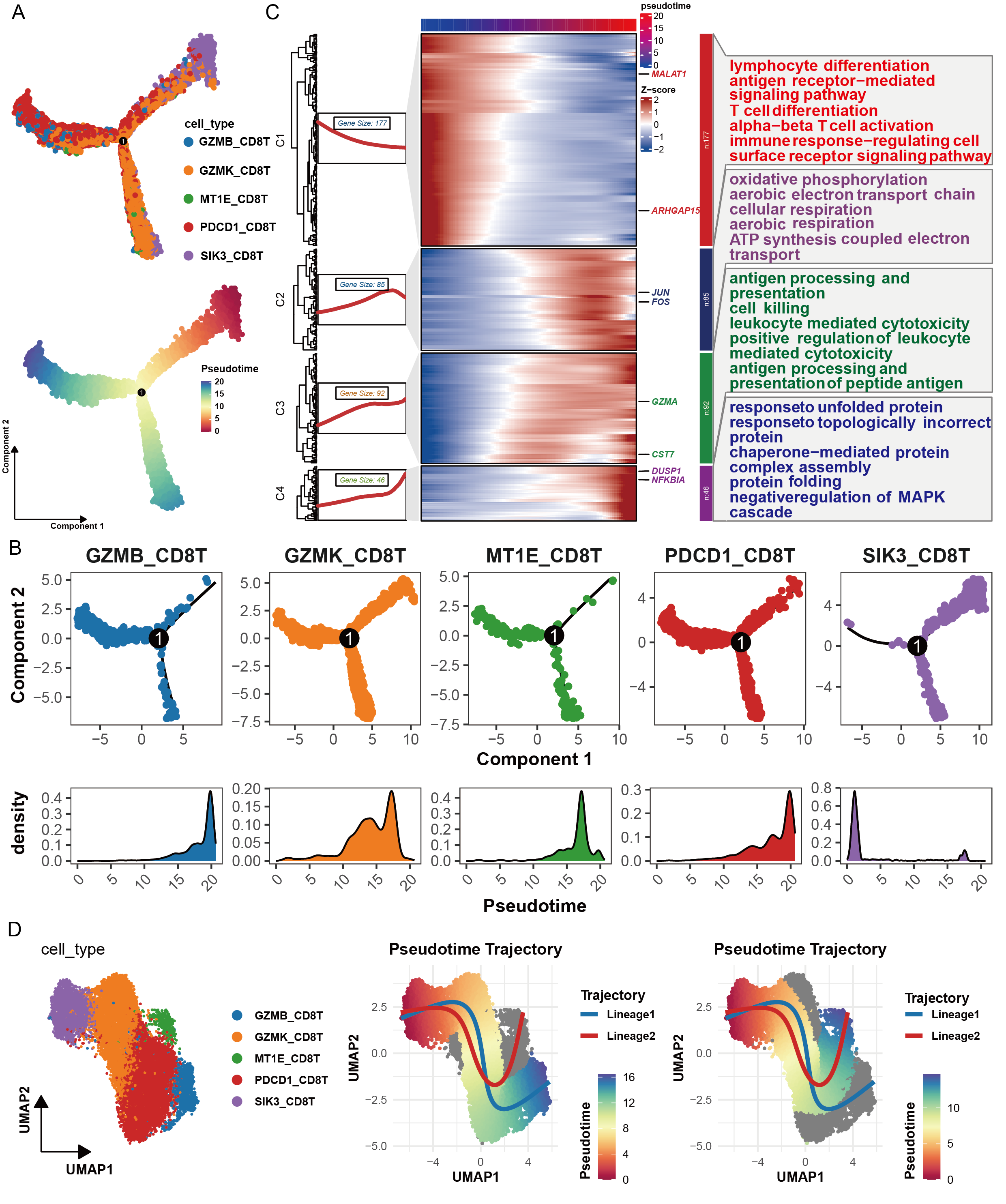


**Supplementary Figure 9. Pseudotime trajectory analyses of CD8^+^ T cell subsets.**

(A) Monocle2-inferred developmental trajectory of CD8^+^ T cell subsets, colored by cell type (top) and pseudotime (bottom).

(B) Branch-dependent mapping of the five CD8⁺ T cell subsets along the developmental trajectory (top) alongside their corresponding density distributions across the pseudotime axis (bottom).

(C) Heatmap of dynamically expressed genes along the pseudotime trajectory, clustered into four gene modules (C1–C4). Representative genes and their associated Gene Ontology (GO) terms or functional pathways are shown for each module.

(D) Slingshot trajectory analysis of CD8^+^ T cell subsets on the UMAP space. Left: UMAP visualization of the five CD8^+^ T cell clusters. Middle and Right: Identification of two distinct differentiation lineages (Lineage 1 and Lineage 2).

Abbreviations: UMAP, Uniform Manifold Approximation and Projection; GO, Gene Ontology.


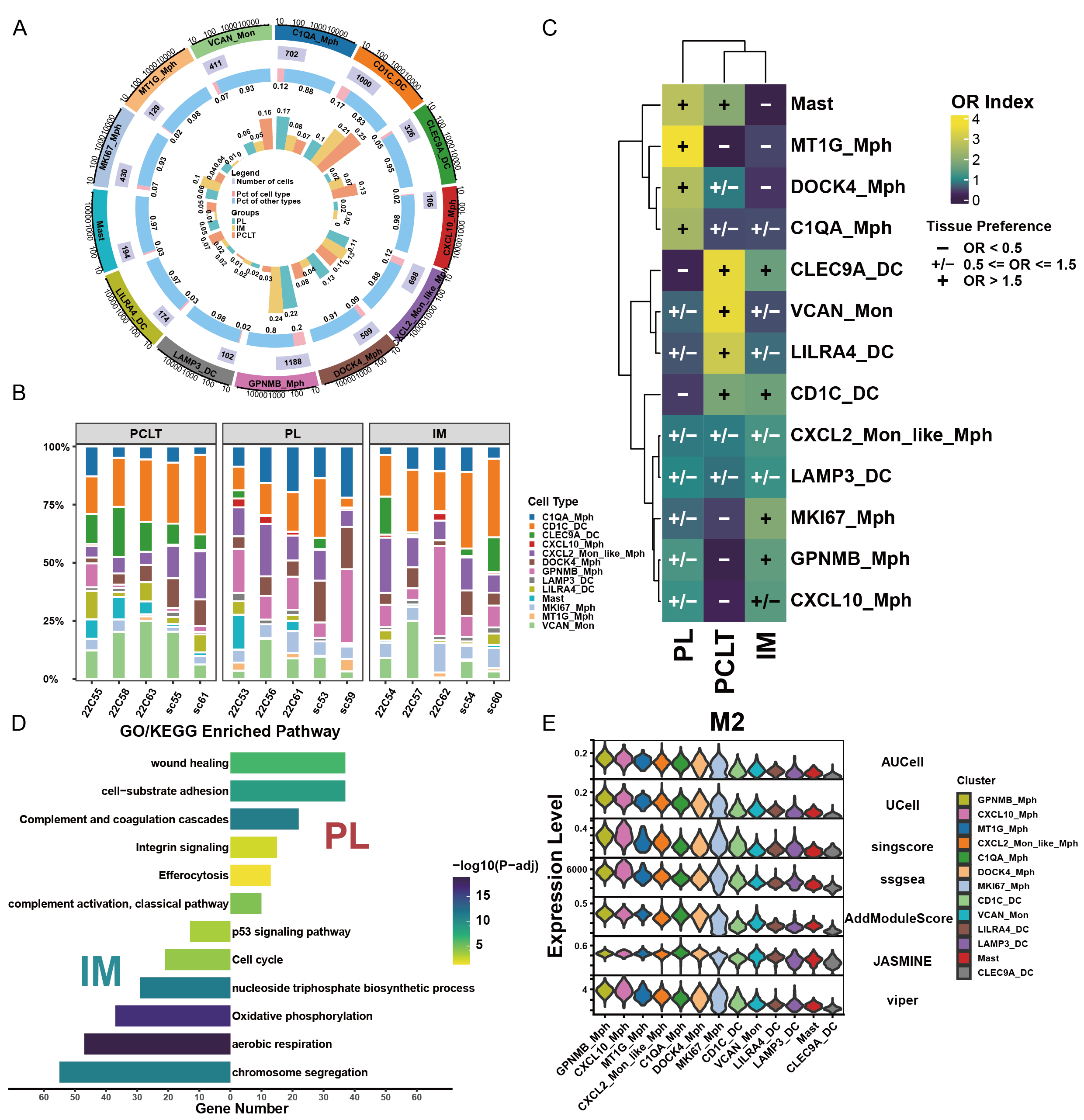


**Supplementary Figure 10. Comprehensive transcriptomic and functional landscape of myeloid cell subsets.**

(A) Multi-circle plot showing cell counts of myeloid cell subsets (outer), their proportions among all cells (middle), and tissue-type-specific cellular composition (inner).

(B) Stacked bar plot showing the proportional distribution of myeloid cell subsets across individual patient samples, stratified by tissue origin (PCLT, PL, and IM).

(C) Heatmap of OR indices for myeloid subsets across the three tissue origins. Odds ratio (OR) > 1.5 indicates enrichment and OR < 0.5 indicates depletion.

(D) Bar chart summarizing the Gene Ontology (GO) biological processes and KEGG pathway enrichment analyses, highlighting representative biological programs and signaling cascades upregulated in PL-derived (top) versus IM-derived (bottom) monocytes/macrophages.

(E) Violin plots comparing M2 signature scores across myeloid cell subsets using various gene-set scoring methods.

Abbreviations: KEGG, Kyoto Encyclopedia of Genes and Genomes; GO, Gene Ontology; PL, primary lesion; IM, intrahepatic metastasis; PCLT, pericarcinomatous liver tissue; OR, odds ratio.


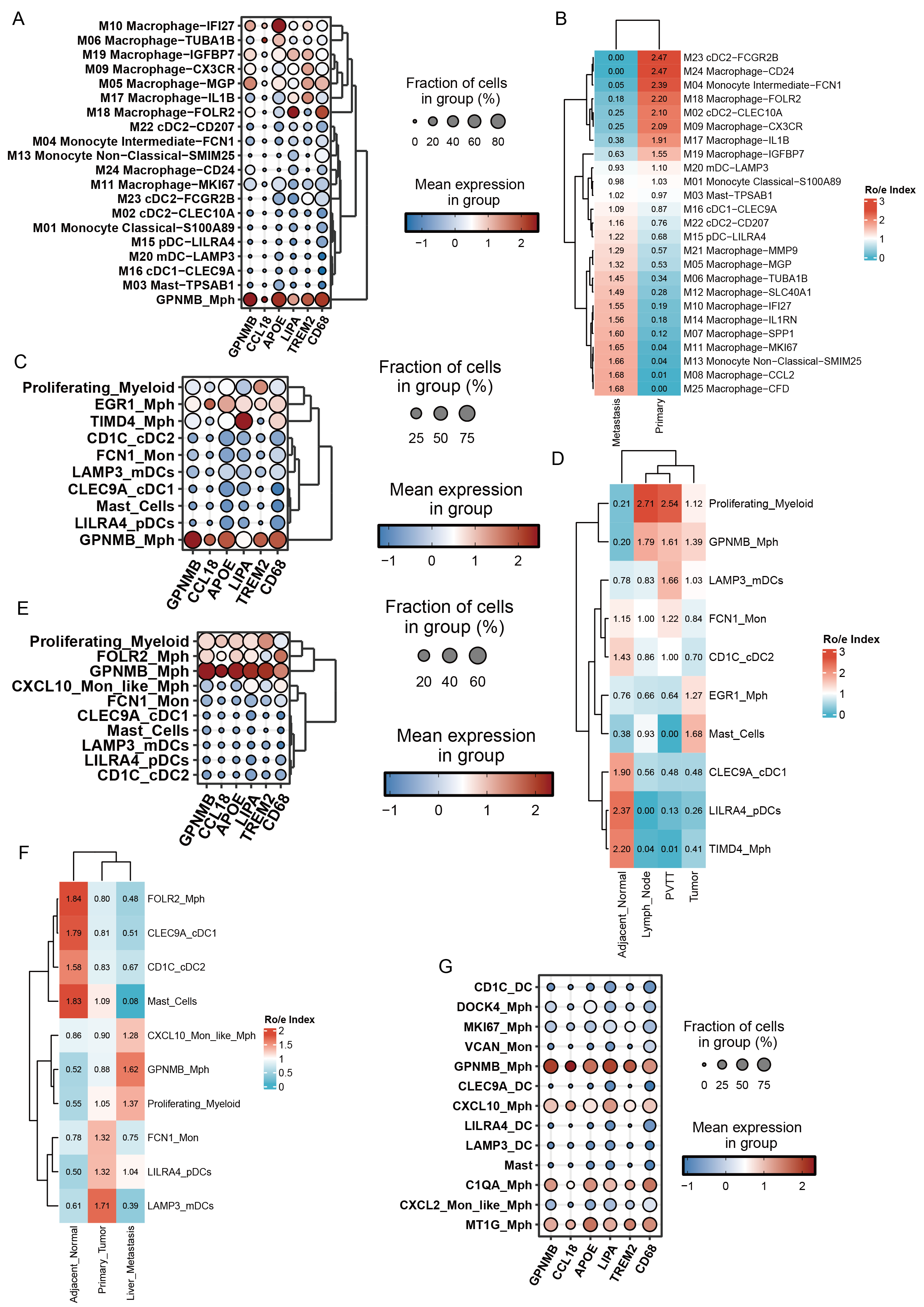


**Supplementary Figure 11. Cross-cohort validation of the transcriptional profile and tissue distribution of GPNMB+ macrophages.**

(A) Dot plot of signature gene expression (GPNMB, CCL18, APOE, LIPA, TREM2, and CD68) across myeloid cell subsets in an external metastatic breast cancer scRNA-seq cohort.

(B) Heatmap of relative over-expectation (Ro/e) indices for myeloid subsets in primary and metastatic lesions from the breast cancer cohort. Ro/e > 1 indicates higher‑than‑expected representation.

(C) Dot plot of signature gene expression across myeloid cell subsets in an external hepatocellular carcinoma with portal vein tumor thrombus (PVTT) cohort.

(D) Heatmap of Ro/e indices for myeloid subsets across adjacent normal tissue, lymph node metastasis, PVTT, and primary tumor in the PVTT cohort.

(E) Dot plot of signature gene expression across myeloid cell subsets in an external colorectal cancer liver metastasis cohort.

(F) Heatmap of Ro/e indices for myeloid subsets across adjacent normal tissue, primary tumor, and liver metastasis in the colorectal cancer cohort.

(G) Dot plot of signature gene expression across myeloid cell subsets within our internal IM-mHCC cohort.

Abbreviations: Ro/e, relative over-expectation; scRNA-seq, single-cell RNA sequencing; PVTT, portal vein tumor thrombus; IM-mHCC, intrahepatic metastatic multifocal hepatocellular carcinoma.


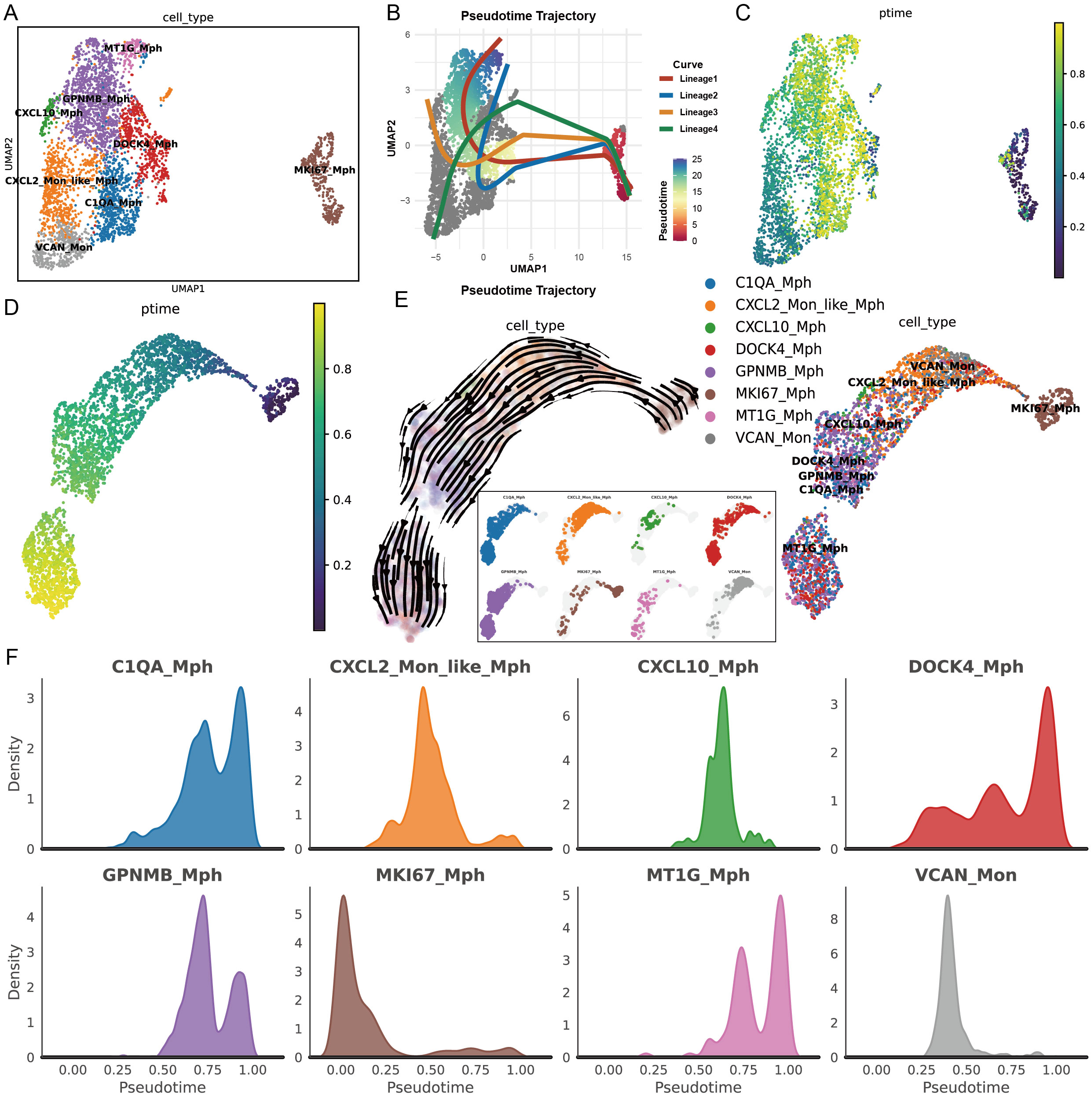


**Supplementary Figure 12. Developmental trajectory of monocyte/macrophage subsets.**

(A) UMAP plot of monocyte/macrophage (Mon/Mph) subsets used for trajectory inference, colored by distinct clusters.

(B) Slingshot trajectory inference. Delineation of four distinct lineages (Lineages 1–4) on the standard UMAP embedding, showing the potential developmental paths of Mon/Mph cells.

(C) Projection of scTour-inferred pseudotime scores onto the original UMAP coordinates.

(D) Mapping of scTour pseudotime scores onto a UMAP embedding derived from the reconstructed scTour latent representation.

(E) scTour vector field analysis illustrating transcriptomic velocity streamlines (arrows) projected onto the latent space. The inset displays the isolated spatial distribution of individual cell clusters across this space.

(F) Density plots illustrating the continuous distribution of individual Mon/Mph subsets along the inferred pseudotime axis.

Abbreviations: Mon/Mph, monocyte/macrophage; UMAP, Uniform Manifold Approximation and Projection.


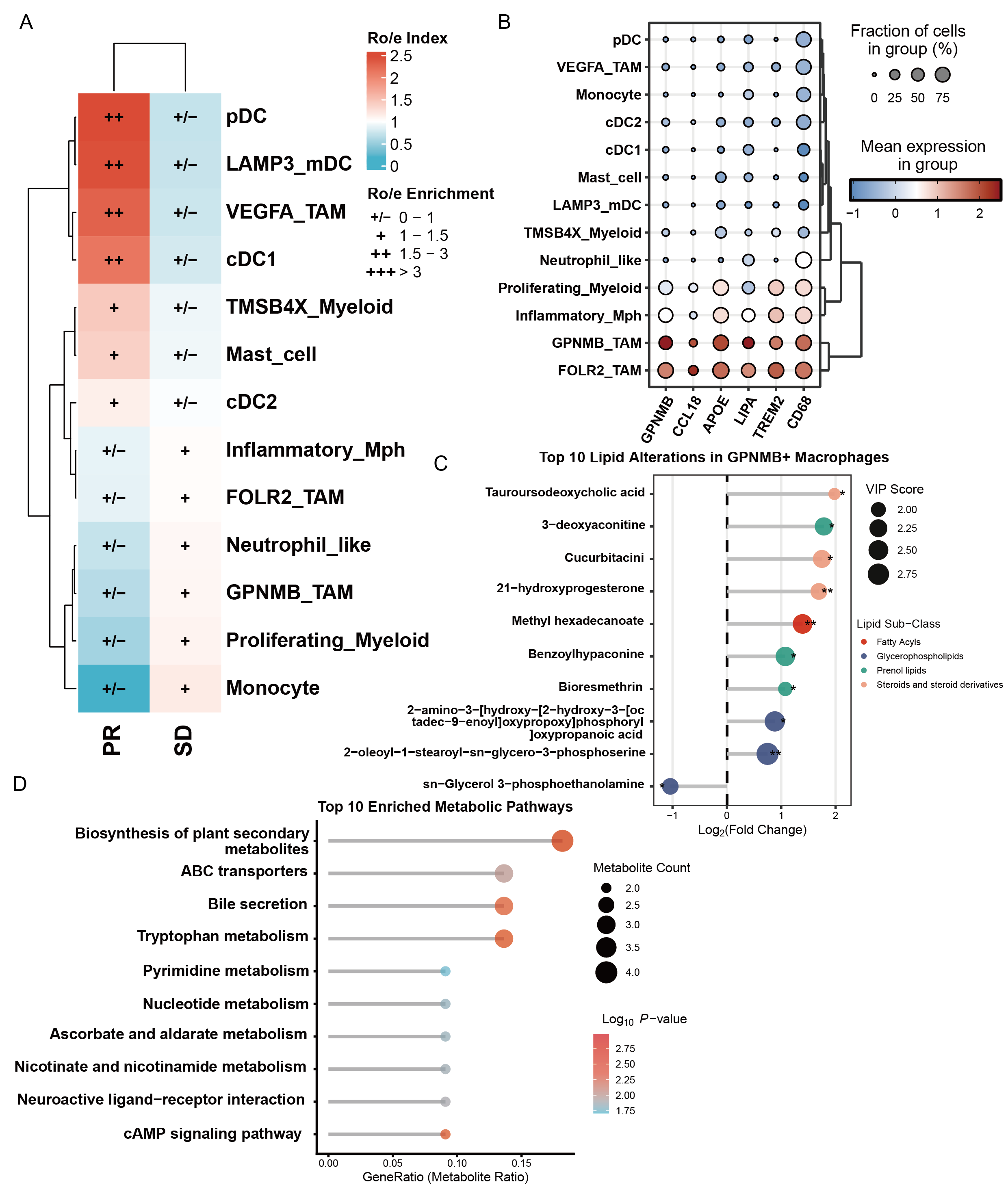


**Supplementary Figure 13. Clinical correlation and multi-omics metabolic profiling of GPNMB^+^ macrophages in independent cohorts.**

(A) Heatmap of relative over-expectation (Ro/e) indices for myeloid subsets in patients exhibiting partial response (PR) versus stable disease (SD) from an independent TNBC immunotherapy scRNA-seq cohort. Ro/e > 1 indicates higher‑than‑expected representation.

(B) Dot plot showing the conserved expression patterns of key defining markers (including GPNMB, CCL18, APOE, LIPA, TREM2, and CD68) across identified myeloid clusters in the TNBC cohort.

(C) Lollipop chart displaying the top 10 differentially abundant lipid-related metabolites between GPNMB-high and GPNMB-low samples from the Zhujiang metabolomics cohort. Node size corresponds to the VIP score, and colors indicate specific lipid sub-classes.

(D) Bubble plot of pathway enrichment analysis based on the differential metabolites.

Abbreviations: TNBC, triple-negative breast cancer; scRNA-seq, single-cell RNA sequencing; PR, partial response; SD, stable disease; Ro/e, relative over-expectation; VIP, variable importance in projection.


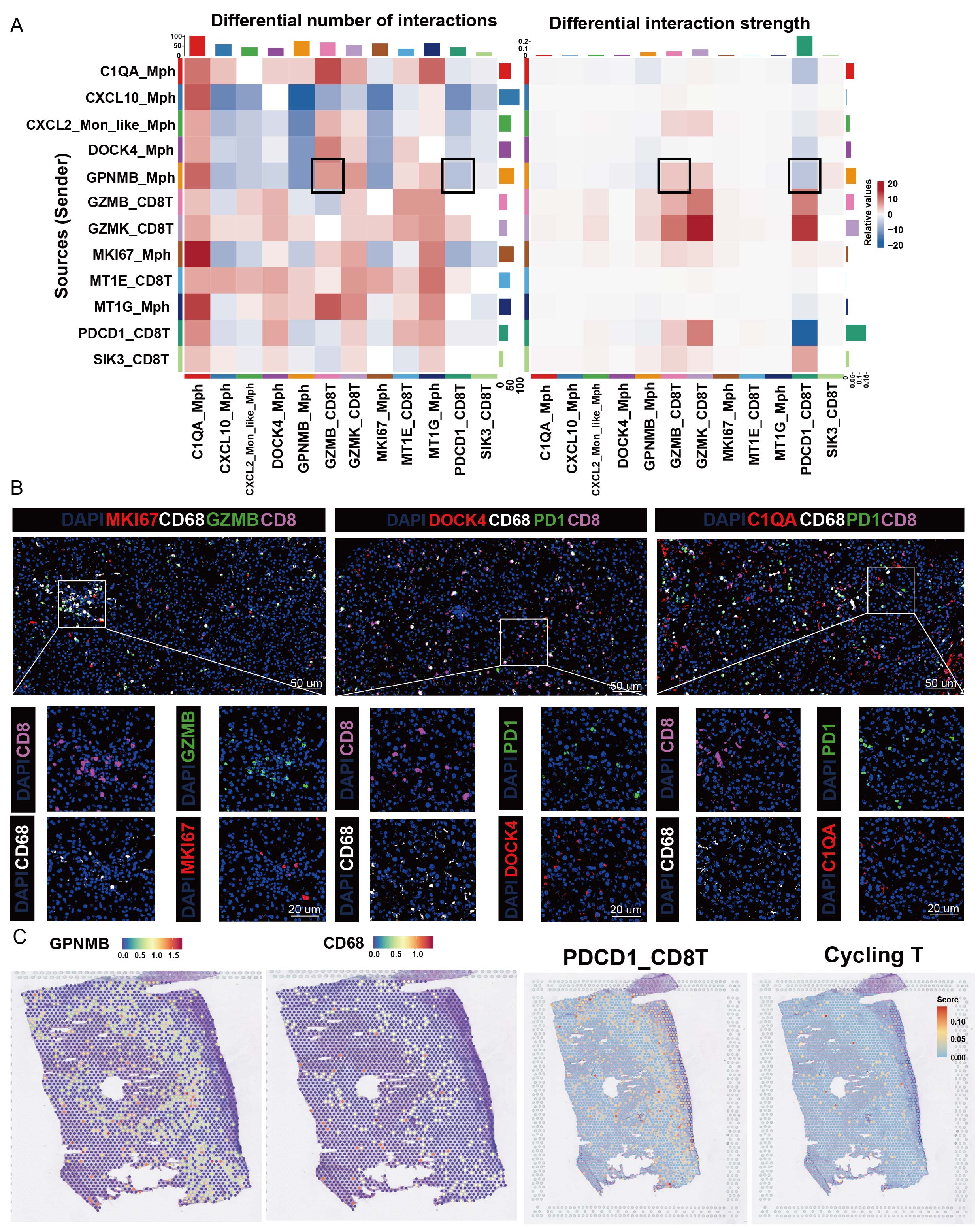


**Supplementary Figure 14. Intercellular crosstalk and spatial architecture in the IM-mHCC tumor microenvironment.**

(A) Heatmaps visualizing the differential interaction frequency (left) and interaction strength (right) between cell populations in the intrahepatic metastasis (IM) compared to the primary lesion (PL), inferred by CellChat. The top and right bar plots quantify the total incoming and outgoing signaling changes for each cell subset, respectively. Bar height corresponds to the degree of alteration in interactions or interaction strength between conditions. The color key represents the relative change: red denotes increased signaling in IM, while blue denotes decreased signaling relative to PL.

(B) Representative mIHC images visualizing specific cellular niches. The panels depict MKI67⁺ macrophages (MKI67⁺CD68⁺) interacting with GZMB⁺ CD8⁺ T cells within the IM (left), alongside DOCK4⁺ macrophages (middle) and C1QA⁺ macrophages (right) interacting with PD-1⁺ CD8⁺ T cells within the PL. Scale bars, 50 μm (main images) and 20 μm (insets).

(C) Spatial transcriptomics (ST) visualization showing spatial expression maps of GPNMB and CD68, along with scores for PDCD1^+^ CD8^+^ T and Cycling T cell signatures.

Abbreviations: IM, intrahepatic metastasis; mIHC, multiplex immunohistochemistry; Mph, macrophage; PL, primary lesion; ST, spatial transcriptomics.


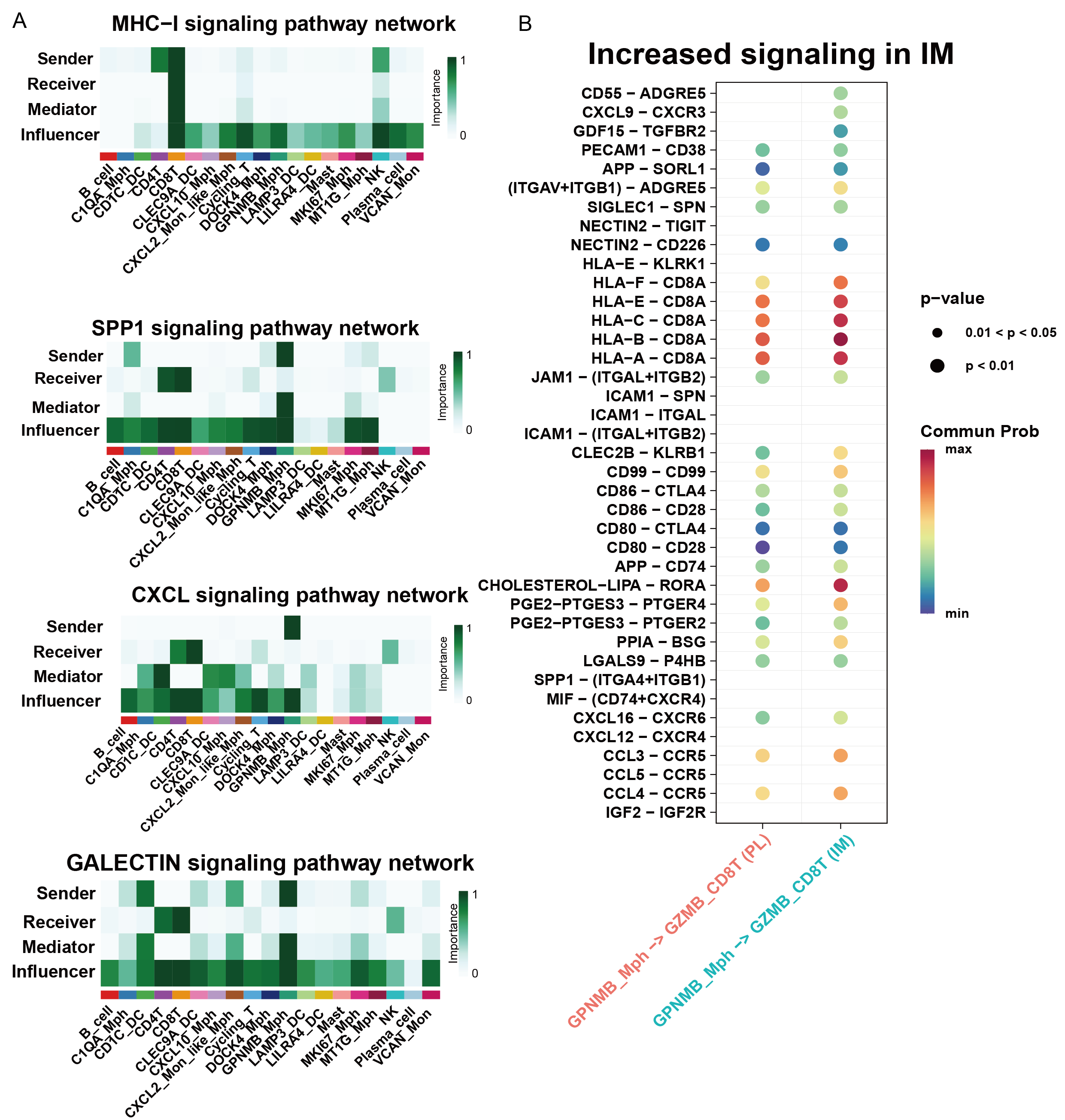


**Supplementary Figure 15. Characterization of specific signaling axes between myeloid and lymphoid subsets.**

(A) Heatmaps identifying the major senders, receivers, mediators, and influencers within the MHC-I, SPP1, CXCL, and GALECTIN signaling networks across myeloid and lymphoid cell subsets.

(B) Bubble plot highlighting significantly upregulated ligand–receptor pairs between GPNMB^+^ Mph and GZMB^+^ CD8^+^ T cells in intrahepatic metastasis (IM) compared to the primary lesions (PL).

Abbreviations: CXCL, chemokine (C-X-C motif) ligand family; GALECTIN, galectin family of proteins; IM, intrahepatic metastasis; MHC-I, major histocompatibility complex class I; Mph, macrophage; PL, primary lesion; SPP1, secreted phosphoprotein 1.


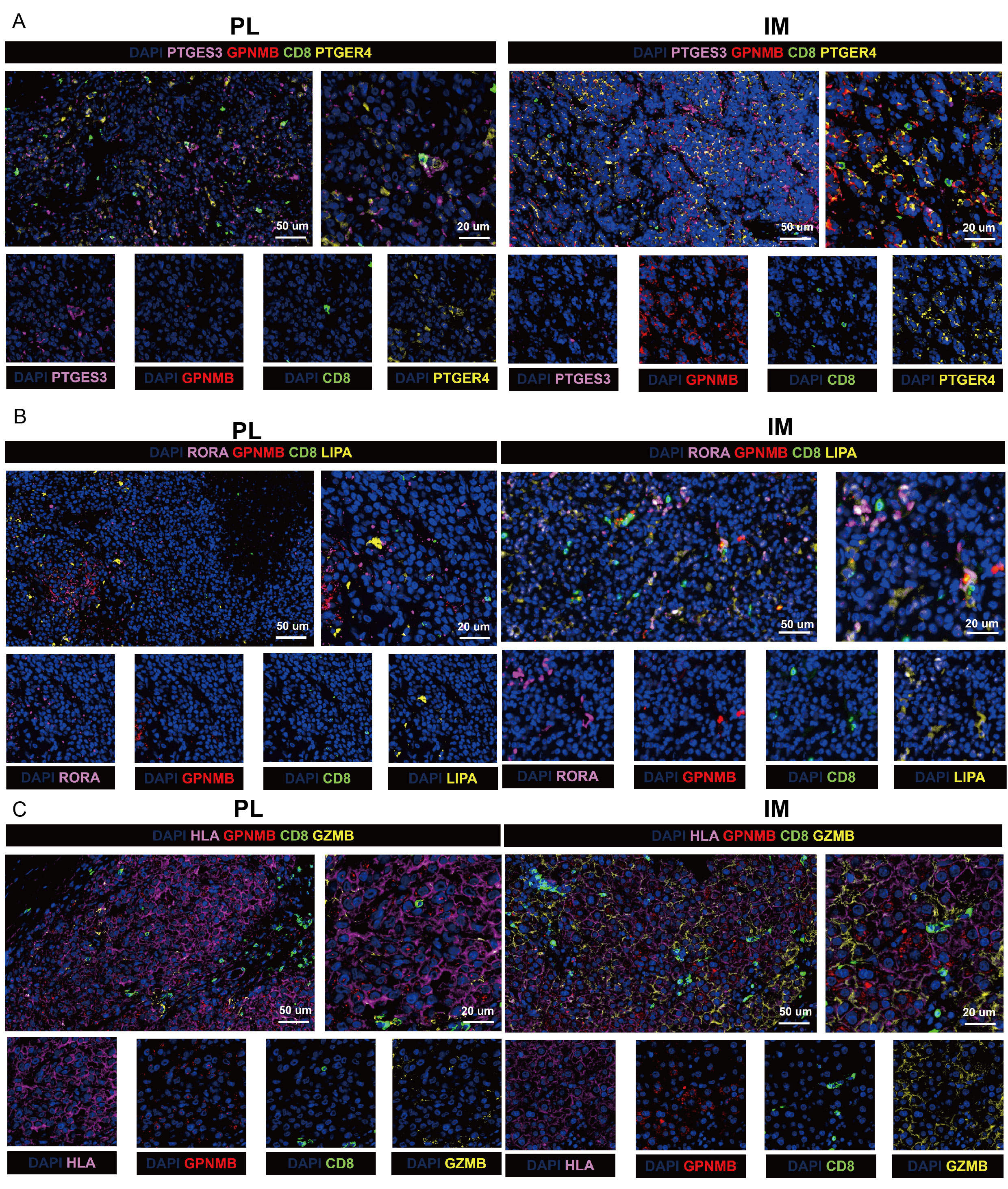


**Supplementary Figure 16. Multiplex immunofluorescence staining of prioritized ligand-receptor pairs in the PL and IM.**

(A) Representative multiplex immunofluorescence (mIF) images illustrating the spatial distribution of PTGES3, GPNMB, CD8, and PTGER4 in the PL and IM.

(B) Representative mIF images illustrating the spatial distribution of RORA, GPNMB, CD8, and LIPA in the PL and IM.

(C) Representative mIF images illustrating the spatial distribution of HLA, GPNMB, CD8, and GZMB in the PL and IM.

For all panels, scale bars represent 50 μm (main overview images) and 20 μm (magnified insets).

Abbreviations: IM, intrahepatic metastasis; mIF, multiplex immunofluorescence; PL, primary lesion.

**
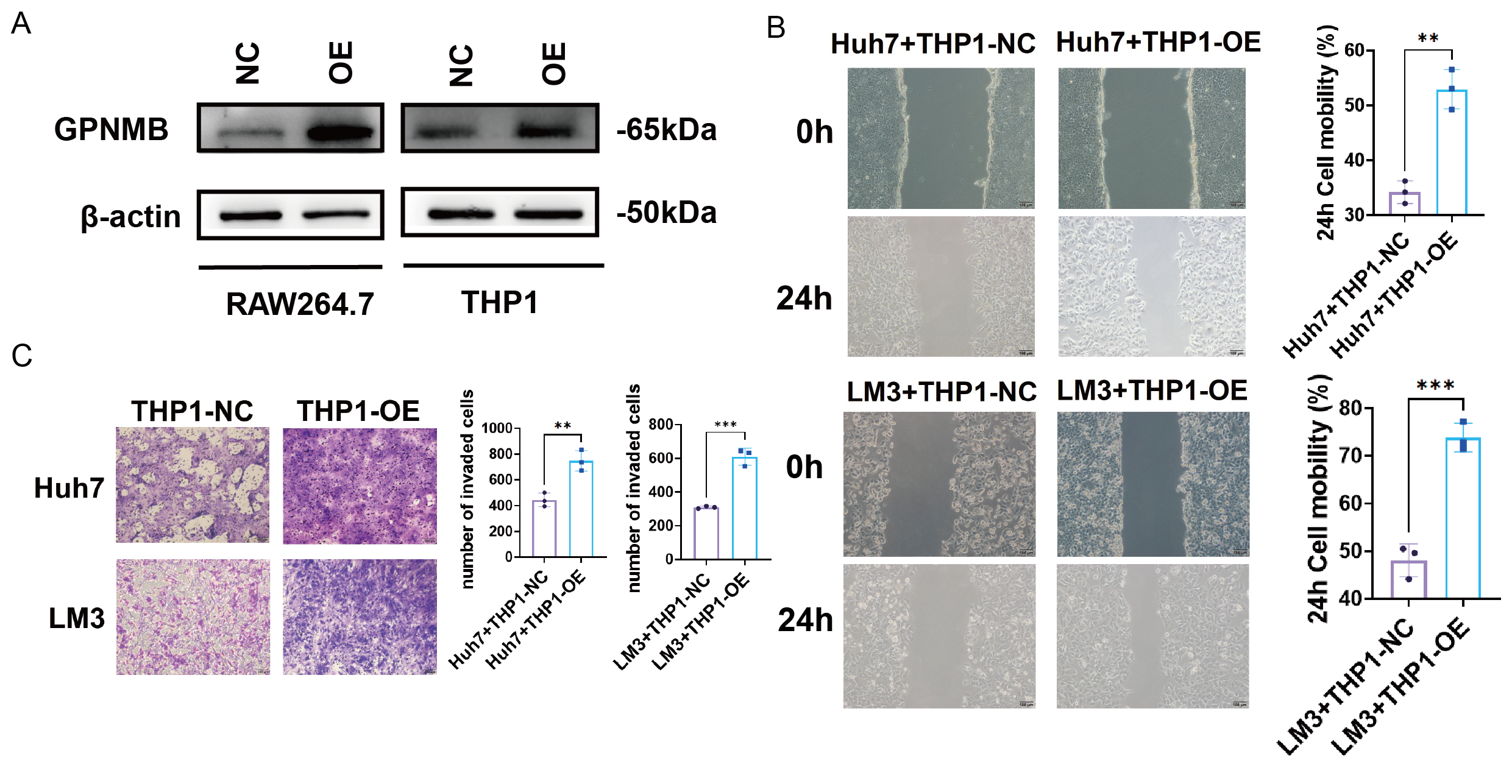
**

**Supplementary Figure 17. *In vitro* validation of GPNMB expression and *in vivo* biosafety assessment of APL_siGpnmb_.**

(A) Western blot analysis of GPNMB protein levels in RAW 264.7 (murine) and THP-1 (human) cells.

(B) Wound healing assay of Huh7/LM3 cells co-cultured with GPNMB⁺ macrophages at 24 h.

(C) Transwell assay of Huh7/LM3 cells co-cultured with GPNMB macrophages.

**
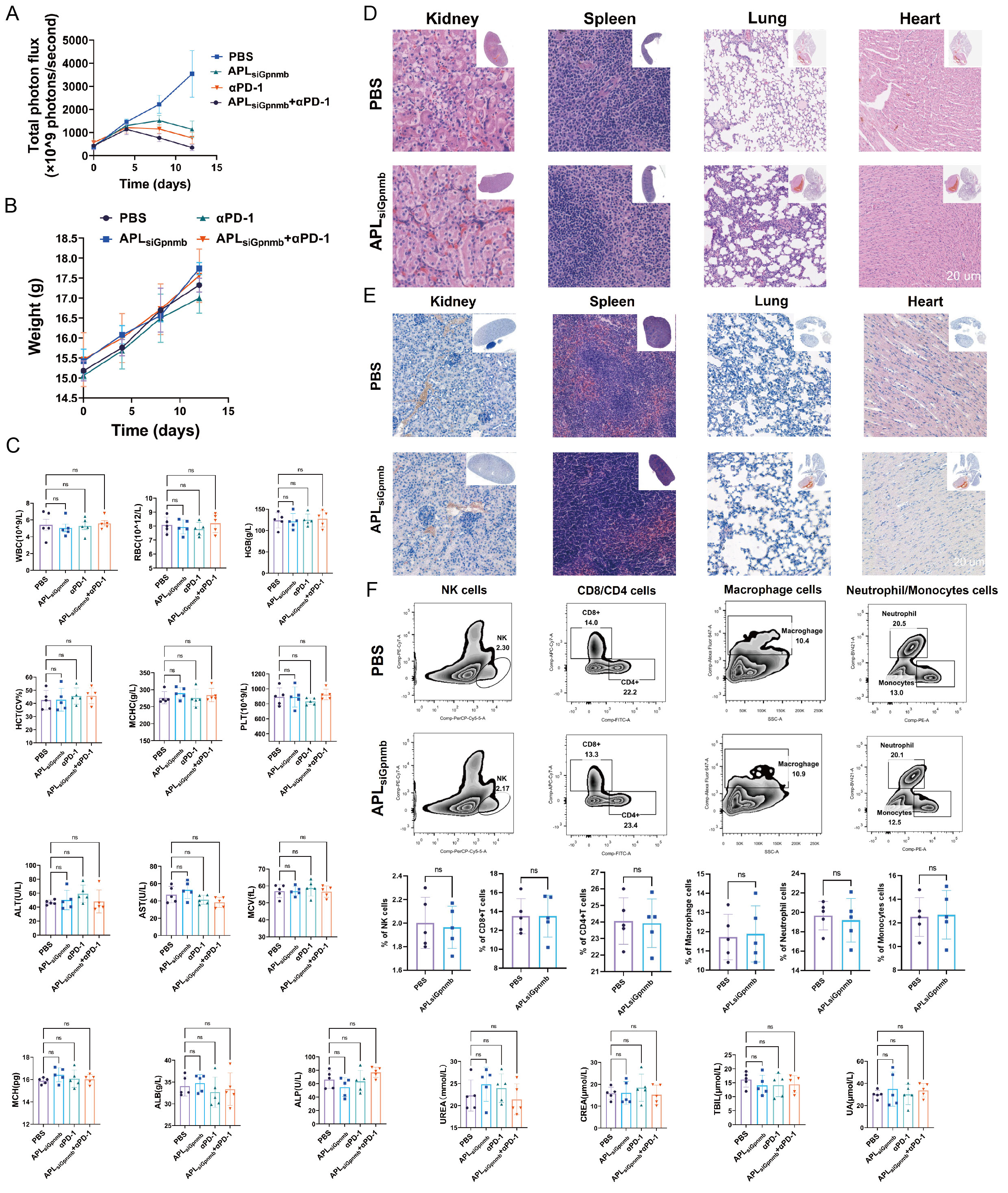
**

**Supplementary Figure 18. *In vivo* biosafety assessment of APL_siGpnmb_.**

(A) Monitoring of tumor progression in the orthotopic HCC mouse model via total photon flux across the four treatment groups.

(B) Body weight changes (g) of mice in the orthotopic tumor model recorded during treatment period.

(C) Complete blood count and serum biochemical analysis of hepatic and renal function biomarkers across all four treatment groups at the experimental endpoint.

(D) Representative H&E-stained histological sections of major organs (kidney, spleen, lung, and heart) harvested from tumor-bearing mice to evaluate short-term structural toxicity (comparing PBS and APLsiGpnmb monotherapy).

(E) Representative histopathological sections of major organs from healthy, immunocompetent mice following a one-month longitudinal safety evaluation to assess delayed systemic toxicity.

(F) Flow cytometric quantification of systemic immune cell populations (NK cells, CD8⁺/CD4⁺ T cells, macrophages, and neutrophils/monocytes) in the spleens of healthy mice following the one-month observation period.

For panels C and E, scale bars represent 20 μm. Data in bar graphs are presented as mean ± SEM. ns, not significant.

Abbreviations: H&E, hematoxylin and eosin; PBS, phosphate-buffered saline; CBC, complete blood count; WBC, white blood cells; RBC, red blood cells; HGB, hemoglobin; HCT, hematocrit; MCHC, mean corpuscular hemoglobin concentration; PLT, platelets; ALT, alanine aminotransferase; AST, aspartate aminotransferase; MCV, mean corpuscular volume; MCH, mean corpuscular hemoglobin; ALB, albumin; ALP, alkaline phosphatase; UREA, blood urea nitrogen; CREA, creatinine; TBIL, total bilirubin; UA, uric acid.
